# Review of global sanitation development

**DOI:** 10.1016/j.envint.2018.07.047

**Published:** 2018-11

**Authors:** Xiaoqin Zhou, Zifu Li, Tianlong Zheng, Yichang Yan, Pengyu Li, Emmanuel Alepu Odey, Heinz Peter Mang, Sayed Mohammad Nazim Uddin

**Affiliations:** a School of Energy and Environmental Engineering, Beijing Key Laboratory of Resource-oriented Treatment of Industrial Pollutants, University of Science and Technology Beijing, Beijing 100083, China; b State Key Joint Laboratory of Environment Simulation and Pollution Control, Research Center for Eco-Environmental Sciences, Chinese Academy of Sciences, 18 Shuangqing Road, Haidian District, Beijing 100085, China; c University of Chinese Academy of Sciences, 19(A) Yuquan Road, Shijingshan District, Beijing 100049, China; d Department of Geography, Faculty of Social Sciences, University of Victoria, PO Box 1700 STN CSC, Victoria, BC V8W 2Y2, Canada

**Keywords:** Sanitation, Bibliometric analysis, Research hotspots, Gaps, Challenges

## Abstract

The implementation of the United Nations (UN) Millennium Development Goals (MDGs) and Sustainable Development Goals (SDGs) has resulted in an increased focus on developing innovative, sustainable sanitation techniques to address the demand for adequate and equitable sanitation in low-income areas. We examined the background, current situation, challenges, and perspectives of global sanitation. We used bibliometric analysis and word cluster analysis to evaluate sanitation research from 1992 to 2016 based on the Science Citation Index EXPANDED (SCI-EXPANDED) and Social Sciences Citation Index (SSCI) databases. Our results show that sanitation is a comprehensive field connected with multiple categories, and the increasing number of publications reflects a strong interest in this research area. Most of the research took place in developed countries, especially the USA, although sanitation problems are more serious in developing countries. Innovations in sanitation techniques may keep susceptible populations from contracting diseases caused by various kinds of contaminants and microorganisms. Hence, the hygienization of human excreta, resource recovery, and removal of micro-pollutants from excreta can serve as effective sustainable solutions. Commercialized technologies, like composting, anaerobic digestion, and storage, are reliable but still face challenges in addressing the links between the political, social, institutional, cultural, and educational aspects of sanitation. Innovative technologies, such as Microbial Fuel Cells (MFCs), Microbial Electrolysis Cells (MECs), and struvite precipitation, are at the TRL (Technology readiness levels) 8 level, meaning that they qualify as “actual systems completed and qualified through test and demonstration.” Solutions that take into consideration economic feasibility and all the different aspects of sanitation are required. There is an urgent demand for holistic solutions considering government support, social acceptability, as well as technological reliability that can be effectively adapted to local conditions.

## Highlights

• Data for sanitation research from year of 1900 to 2016 have been collected and analyzed.
• Hotspots for sanitation research have been discussed for future work based on the keywords data.
• The constraints for global sanitation have been identified.
• Promising technologies have been evaluated and proposed.

## Graphical abstract

**Figure:**
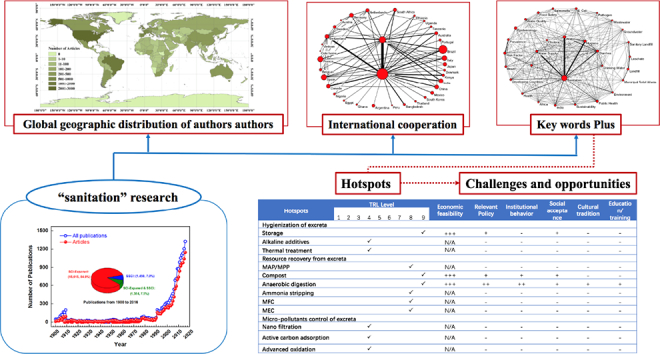

## 1 Introduction

The United Nations (UN), many local governments, and international organizations have launched programs to deal with the negative impact on human health and the environment caused by the lack of access to adequate sanitation. In the 1990s, 192 UN member states and at least 23 international organizations agreed to the Millennium Development Goals (MDGs) at the World Summits. MDG 6 (Target 10) was intended to halve the proportion of people without sustainable access to safe drinking water and basic sanitation by 2015. In September 2015, the 2030 Agenda for Sustainable Development Goals (SDGs) was adopted by world leaders at the UN, which calls on countries to begin new efforts to achieve 17 SDGs over the next 15 years, including the goal to “ensure the availability and sustainable management of water and sanitation for all”. To inspire action to tackle the global sanitation crisis, “World Toilet Day”, which was established by the World Toilet Organization in 2001, was declared an official UN holiday in 2013. Every November 19th since then, UN-Water, local civil society organizations, and volunteers have planned events all over the world with themes such as “Toilets and Nutrition”, “Toilets and Jobs”, and “Wastewater”. Narendra Modi, the prime minister of India who launched projects like the Swachh Bharat Mission (Clean India Mission) in 2014, forged ahead to eliminate open defecation with the goal of constructing toilets in every household in the country by 2019. India is making impressive headway with its comprehensive planning for achieving this goal (Liangyu, 2017). President Xi Jinping proposed a “toilet revolution” in China's rural areas in 2015. Thereafter, the China National Tourism Administration (CNTA) quickly started a “toilet revolution in tourism”, and great efforts have been undertaken to promote better sanitation (Cheng et al., 2018). More than 68,000 public toilets have been refurbished in China (Wong, 2017). Global sanitation efforts have been undertaken by governments as well as non-governmental organizations, where there are additional resources and manpower. In 2011, the Bill and Melinda Gates Foundation (BMGF) launched a research program named “Reinvent the Toilet Challenge (RTTC)”, which aimed to build sustainable and financially-profitable sanitation services and businesses that operate in poor, urban settings in both developed and developing nations. The new toilet system that won the challenge is a truly aspirational next-generation product that operates “off the grid” without connections to centralized water, sewers, or electrical supplies, removes harmful organisms from human waste, and recovers valuable resources such as energy, clean water, and nutrients. The toilet also costs (consists of both fundamental investment and operation cost) <5 cents (US) per user per day. In 2013, after the successful implementation of worldwide activity in 2011 and 2012, the BMGF expanded the project by supporting for regional programs, including “Reinvent the Toilet Challenge – India (RTTC-India)” and “Reinvent the Toilet Challenge – China (RTTC-China)”, encourages researchers and institutions to innovate and design new-generation toilets in the China and India locally, but the RTTC-China also accepted the proposal which was proposed by Chinese leading team cooperated with the foreign partners.

However, although the percentage of people gaining access to improved sanitation increased from 54% to 68% and the percentage for open defecation has fallen from 24% to 13%, the world still missed the MDG target (WHO, 2015). Currently, there are still 4.5 billion people lacking safely managed sanitation, and among them, 2.3 billion still do not have basic sanitation services. This number includes 600 million people who share a toilet or latrine with other households and 892 million people – primarily in rural areas – who defecate in the open, as reported by the World Health Organization (WHO) and the UN International Children's Emergency Fund (UNICEF) on July 12th, 2017 (Osseiran et al., 2017). WHO defines sanitation as the provision of facilities or services that separates people from urine and feces. Safe access to sanitary toilets and the management of excreta are the basic targets for global sanitation, and these targets are important to realizing effective resource recycling. Resource recycling, generally called “sustainable sanitation”, should be economically viable, socially acceptable, and technically and institutionally appropriate. It should protect the environment and conserve natural resources. Developing economical, acceptable, technically flexible, and environmentally-friendly sanitation technology for the next generation requires research into sanitation technologies development.

Here we present a statistical analysis based on published research that appeared in journals between 1992 and 2016 intended to identify trends in publication, explore research patterns and hotspots, isolate the specific issues with global sanitation as well as the assessments of those issues. The results offer a comprehensive overview of current issues and perceptions in sanitation research. This work can also help researchers develop ideas concerning future research areas and make more informed decisions.

## 2 Materials and methods

### 2.1 Data sources

Information about scientific output was extracted from the Science Citation Index EXPANDED (SCI-EXPANDED), Social Sciences Citation Index (SSCI), and the 2017 Journal Citation Reports (JCR), Science Edition, from Clarivate Analytics on August 17, 2017. The 2017 JCR covers 11,459 journals across 236 scientific disciplines spanning 81 countries. In this study, we searched for the keywords “sanitation” or “sanitary” in the period from 1900 to 2016.

### 2.2 Data analysis

We used bibliometric analysis, which has been adopted in other studies (Chiu et al., 2004, Fu et al., 2013, Zheng et al., 2018) to investigate sanitation research trends worldwide. We utilized numerous markers to help identify trends in publication, such as the document type, language, categories, and journals, as well as countries/territories, institutions, and the *h*-index. It has to be mentioned that h-index was regarded as the h of Np articles were cited no less than h times each and the other (Np-h) articles were cited no more than h times each (Hirsch, 2005). The document type, language, output, subject category, journal, country, institute, source title, abstract keywords, and *h*-index were all analyzed using Microsoft Excel 2010. The frequency analysis was conducted using BibExcel 1.0.0.0 (Persson et al., 2009). We used BibExcel for the co-occurrence analysis and Pajek 1.0.0.1 (Nooy et al., 2011) network diagrams for the cooperation analysis. By identifying the institution and country of at least one author, we were able to estimate the influence of a country and its research facility. Research from Hong Kong was grouped with that of China (Chuang et al., 2011), and works from England, Scotland, Northern Ireland, and Wales were included with research from the UK. We assigned the tag of “internationally collaborative publication” to works that had authors from multiple countries. We identified articles as being the “independent type” when the researchers were all from the same location. We used the tag “single institute publication” when the researchers' addresses were all from the same research facility, and “inter-institutionally collaborative publications” identified works from the authors from different research facilities (Fu et al., 2012). We used data from the JCR, Science Edition, 2017 for the values of the journal impact factor (JIF). We also applied “word cluster analysis” (Mao et al., 2010) to explore the research patterns and hotspots. This analysis encompassed the distribution of author keywords, article title, article abstracts, and the KeyWords Plus during the time period, so that we were able to isolate the specific research trends with global sanitation as well as the assessments of those issues.

## 3 Sanitation activities in scientific research

### 3.1 Publication patterns

#### 3.1.1 Characteristics of publication outputs

**Fig. 1:**
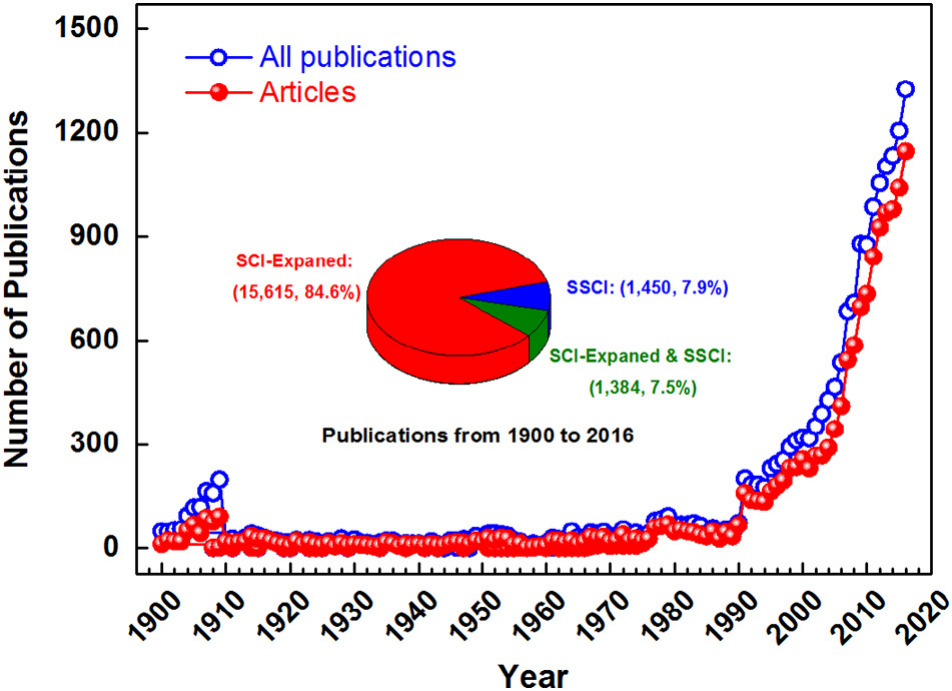
Growth trend of the SCI-Expanded or SSCI publications on “sanitation” research over the last 116 years.

We identified 18,449 publications related to sanitation research in the SCI-Expanded and SSCI databases between 1900 and 2016, and we found a continual growth trend, as shown in Fig. 1. There were 15,615 (84.6%) papers indexed in the SCI-Expanded and 1450 (7.9%) papers indexed in the SSCI on sanitation; 1384 (7.5%) were indexed in both the SCI-Expanded and SSCI. Fig. 1 shows that after 1991, the number of publications rose significantly.

More than 90% of articles in Web of Science have, since 1991, included abstracts, compared with only 20% articles in 1990 (Ho et al., 2010), 14,645 papers from 1992 to 2016 were selected as study samples. We grouped these works into 17 types of documents. Articles comprised 81.6% (11,956) of the total number of works examined, making them the most common document type. The remaining publications consisted of proceedings (981), reviews (954), editorial material (262), meeting abstracts (176), book reviews (74), news items (73), letters (64), book chapters (44), corrections (19), notes (18), reprints (8), discussions (6), items about an individual (4), biographical items (3), retracted publications (2), and retractions (1). We did not use any other document types in this study because articles were the most common type of work. Of the 11,956 articles, 10,049 (84%) were published in English, followed by Portuguese (651), Spanish (508), French (315), German (134), Japanese (65), Italian (62), Polish (61), Russian (29), Chinese (16), Croatian (14), Czech (9), Turkish (7), Hungarian (6), Korean (6), Slovene (5), Dutch (4), Romanian (4), Serbian (4), Slovak (3), Malay (2), Lithuanian (1) and Serbo-Croatian (1).

**Fig. 2:**
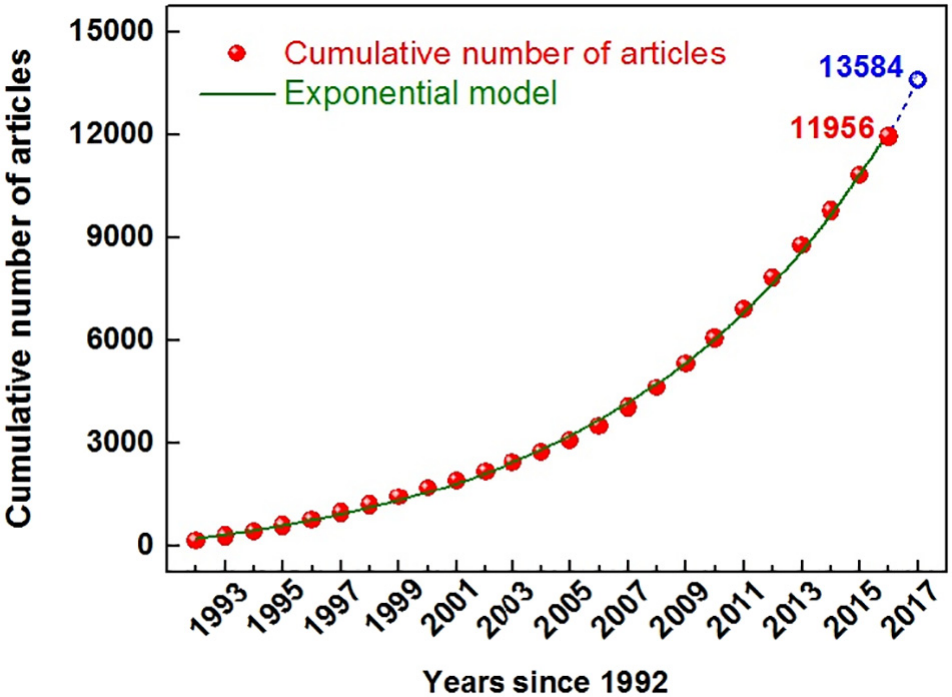
The variation in the cumulative number of articles since 1992.

We found that the number of articles published per year increased over the study period. There were 140 articles published in 1992 and 1147 in 2016. We created an exponential model of the cumulative annual number of articles (Fig. 2).

$$C=-816.17+915.50e^{0.10\text{5Y}}\;\left(R^{2}>0.999\right)$$

In this model, C is the cumulative number of articles. Y is the number of years that have passed since 1992. We used this model to identify the total number of articles for a given time period. From 1992 to 2017, our model showed that 13,584 texts were published. The quantity published in 2021 was predicted to be 2212. In 2016, 1147 works were published, which is about half the number predicted for 2021.

The mean number of authors per article grew from 3.5 in 1992 to 6.0 in 2016. This was an increase of 72.4% (Table 1). However, this increase was dwarfed by the 122.8% rise in the number of institutions per article. There was a similar increase in the mean number of references per article, 261.0%. The average article length and average number of countries per article fluctuated slightly, with an overall mean length of 9.1 pages and average of 1.2 countries. The number of times each article was cited fluctuated, with a peak average of 27.4 citations in 1997 and an average low of less than five citations in recent years. Articles most frequently had three authors; 2153 articles (18.0%) had three authors, then two and four authors accounted for 1882 (15.7%) and 1879 (15.7%) of the total, respectively. About 10% of the sample consisted of five (1491, 12.4%), one (1463, 12.2%), and six (1136, 9.5%) authors together. The *Lancet* published the article with the largest number of authors (722). These names all appeared on the article “Global, regional, and national comparative risk assessment of 79 behavioral, environmental and occupational, and metabolic risks or clusters of risks in 188 countries, 1990–2013” (Forouzanfar et al., 2015).

**Table 1:** Article output from 1992 to 2016.

Year	TA	AU	AU/TA	NR	NR/TA	PG	PG/TA	NI	NI/TA	NC	NC/TA	TC	TC/TA
1992	140	490	3.5	1535	11.0	1051	7.5	192	1.4	141	1.0	1810	12.9
1993	138	430	3.1	2062	14.9	1061	7.7	201	1.5	151	1.1	1918	13.9
1994	135	421	3.1	1693	12.5	1022	7.6	184	1.4	149	1.1	1973	14.6
1995	164	597	3.6	2596	15.8	1216	7.4	259	1.6	180	1.1	2515	15.3
1996	181	646	3.6	4044	22.3	1413	7.8	299	1.7	205	1.1	3777	20.9
1997	196	659	3.4	4409	22.5	1530	7.8	301	1.5	210	1.1	5367	27.4
1998	232	816	3.5	5119	22.1	1984	8.6	384	1.7	251	1.1	3333	14.4
1999	235	768	3.3	5489	23.4	2081	8.9	386	1.6	264	1.1	4012	17.1
2000	257	851	3.3	6385	24.8	2266	8.8	406	1.6	283	1.1	5537	21.5
2001	231	792	3.4	5077	22.0	2061	8.9	367	1.6	265	1.1	4837	20.9
2002	267	985	3.7	7509	28.1	2687	10.1	500	1.9	340	1.3	6674	25.0
2003	268	945	3.5	7162	26.7	2457	9.2	470	1.8	313	1.2	5574	20.8
2004	290	1085	3.7	7901	27.2	2669	9.2	544	1.9	366	1.3	6458	22.3
2005	345	1295	3.8	8828	25.6	3173	9.2	641	1.9	414	1.2	5856	17.0
2006	411	1674	4.1	10,925	26.6	3891	9.5	811	2.0	513	1.2	5784	14.1
2007	543	2239	4.1	15,228	28.0	4926	9.1	1088	2.0	661	1.2	8525	15.7
2008	587	2365	4.0	16,905	28.8	5635	9.6	1170	2.0	747	1.3	7657	13.0
2009	697	2942	4.2	20,099	28.8	6286	9.0	1457	2.1	872	1.3	8704	12.5
2010	735	3094	4.2	23,183	31.5	6906	9.4	1551	2.1	947	1.3	7521	10.2
2011	842	3680	4.4	28,299	33.6	8297	9.9	1864	2.2	1102	1.3	8454	10.0
2012	927	4379	4.7	32,783	35.4	9025	9.7	2164	2.3	1280	1.4	10,634	11.5
2013	969	4505	4.6	36,103	37.3	9818	10.1	2328	2.4	1386	1.4	7064	7.3
2014	979	4847	5.0	37,314	38.1	10,005	10.2	2482	2.5	1429	1.5	4994	5.1
2015	1040	6102	5.9	40,388	38.8	10,994	10.6	3091	3.0	1631	1.6	3075	3.0
2016	1147	6920	6.0	45,405	39.6	12,698	11.1	3505	3.1	1852	1.6	1244	1.1
Total	11,956	53,527		376,441		115,152		26,645		15,952		133,297	
Average			4.0		26.6		9.1		1.9		1.2		14.7

Note: TA: quantities of articles; AU: quantities of authors; NR: cited reference counts; PG: page counts; NI; institute counts; NC; country counts; TC: number of times cited; and AU/TA, NR/P, PG/TA, NI/TA, NC/TA, and TC/TA: mean number of authors, pages, references, institutes, countries, times cited, per article.

The *Lancet's* publication entitled “A comparative risk assessment of the burden of disease and injury attributable to 67 risk factors and risk factor clusters in 21 regions, 1990–2010: A systematic analysis for the Global Burden of Disease Study 2010” (Lim et al., 2012) had 207 contributors from 26 countries and 103 institutes, which reviewed the leading risk factors for global disease burden. The researchers presented evidence of key risks, including poor water quality and sanitation, vitamin A and zinc deficiencies, and ambient particulate matter pollution. The second most cited article, “Global mortality, disability, and the contribution of risk factors: Global Burden of Disease Study” (Murray and Lopez, 1997) with 2161 citations, was published in *Lancet* by WHO, Switzerland, and Harvard University in 1997. The article “Global, regional, and national comparative risk assessment of 79 behavioral, environmental and occupational, and metabolic risks or clusters of risks in 188 countries, 1990–2013: A systematic analysis for the Global Burden of Disease Study 2013” (Forouzanfar et al., 2015) was also published in *Lancet* by 722 authors from 325 institutes in 81 countries; it was cited 306 times within one year. It ranked second for the most frequently cited research article per year. This research emphasized the risks from unsafe sanitation and water; it disclosed that behavioral, environmental, occupational, and metabolic risks can explain half of the global mortality rate and more than one-third of the global-disability-adjusted life-years (DALYs), indicating that there were many opportunities for prevention. The three most cited publications showed that sanitation is closely linked to health conditions for humans; poor sanitation and bad hygiene result in the spread of disease. Thus, improving sanitation is key to creating a better quality of life for millions of people.

#### 3.1.2 Output in subject journals and categories

**Fig. 3:**
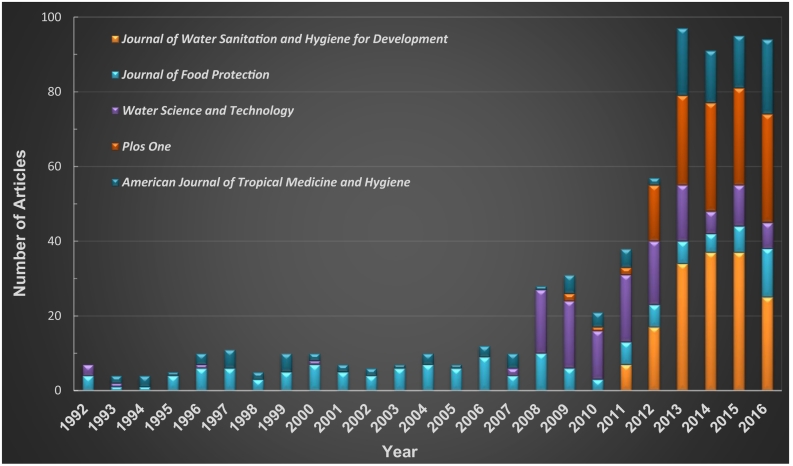
Growth tendency of the 5 most productive journals (TA > 120).

The 11,956 articles we utilized for our analyses came from a wide range of journals. They appeared in 2714 publications that dealt with sanitation issues. These articles addressed a broad range of topics, including 143 subject categories in the SCI-Expanded and SSCI databases. We found that 1306, or almost half (48.1%), of the journals investigated had only one article that appeared in our results. Other journals had more articles (in some cases, many more). Of the remaining journals, 443 (16.3%) had two articles and 286 (10.5%) journals had three articles. About 5% of the journals (141 publications) had 4 articles, while 227 (8.4%) journals published 10 or more. Table 2 shows our data for the top 20 most productive journals (TP > 57). This table includes the ISI category as well as the position of the journal within its category. Table 2 also includes a summary of the JIF, *h*-index, and journal's country of origin. The top 20 most productive journals were extremely influential. The number of their published articles comprised about 15.8% of the works we investigated. The *Journal of Water Sanitation and Hygiene for Development*, with an *h*-index of 8, published the most articles (157; 1.3%), followed by the *Journal of Food Protection* (140; 1.2%), *Water Science and Technology* (130; 1.1%), *PLOS ONE* (128; 1.1%), and *American Journal of Tropical Medicine and Hygiene* (122; 1.0%). There were some unusual results in our analysis. For example, *Water Research* had the highest JIF value (6.942) and *h*-index (33); this journal was the top publication for water resources (Q1: 1/88). However, it came in eighth place in our results for its output. The *Journal of Water Sanitation and Hygiene for Development* is a journal of the International Water Association (IWA), which emphasizes issues of concern in developing and middle-income countries, as well as in disadvantaged communities worldwide. The journal articles address themes that explore the water supply, sanitation, and hygiene, which potentially explains why this is the most productive journal. There were various trends exposed by the relationship between the *h*-index, JIF, and the rank order of the 20 most productive journals. We found that *Water Science and Technology* ranked third among the most productive journals. However, this journal's JIF (1.197) came in 16th place among the top 20 publications. In addition, its *h*-index (12) only ranked 17th. We found that the 14th-ranked journal (*Environmental Science & Technology)* had a JIF of 6.198, which placed it second among the other journals. Its *h*-index was 27, which then moved it to third place. This interesting pattern may be partly due to the fact that the highest impact journals are not as specialized as those with less impact. In addition, with their stronger impact, they may target higher quality studies or more often consider research that offers an original approach to a problem or idea. There was a clear supremacy of publications that came from developed nations: the UK published nine influential journals, and seven publications were from the USA. Brazil had a mere two journals, which was mirrored by the Netherlands (2). There was a strong dominance of five journals. These publications were responsible for 677 (5.6%) of the articles studied. Fig. 3 shows our results for the trends at these top five publications.

**Table 2:** The performance of the top 20 most productive journals (1992–2016).

No	Journal	TA (%)	ISI category and position	JIF (R)	*h*-index (R)	*h*-index/TP % [R]	Journal country
1	Journal of Water Sanitation and Hygiene For Development	157 (1.31)	Water Resources (Q4: 73/88)	0.688 (19)	8 (18)	5.10 (19)	USA
2	Journal of Food Protection	140 (1.17)	Biotechnology & Applied Microbiology (Q3: 115/158)	1.417 (15)	25 (4)	17.86 (12)	USA
			Food science & technology (Q3: 65/129)				
3	Water Science and Technology	130 (1.09)	Engineering, Environmental (Q4: 38/49)	1.197 (16)	12 (17)	9.23 (17)	England (UK)
			Environmental Sciences (Q3: 169/229)				
			Water Resources (Q3: 61/88)				
4	Plos One	128 (1.07)	Multidisciplinary sciences (Q1: 15/64)	2.806 (9)	17 (11)	13.28 (16)	USA
5	American Journal of Tropical Medicine and Hygiene	122 (1.02)	Public, environmental & occupational health (Q2: 48/176) Tropical medicine (Q2: 5/19)	2.549 (10)	28 (2)	22.95 (8)	USA
6	Ciencia & Saude Coletiva	109 (0.91)	Public, environmental & occupational health (Q4: 134/157*)	0.780 (18)	7 (19)	6.42 (18)	Brazil
7	BMC Public Health	100 (0.84)	Public, environmental & occupational health (Q2: 63/176)	2.265 (11)	17 (11)	17.00 (13)	England (UK)
8	Water Research	96 (0.80)	Engineering, Environmental (Q1: 2/49)	6.942 (1)	33 (1)	34.38 (2)	England (UK)
			Environmental Sciences (Q1: 8/229)				
			Water Resources (Q1: 1/88)				
9	Food Control	91 (0.76)	Food science & technology (Q1: 12/129)	3.496 (7)	18 (10)	19.78 (10)	England (UK)
10	Waste Management	90 (0.75)	Engineering, Environmental (Q1: 12/49)	4.030 (4)	24 (5)	26.67 (5)	USA
			Environmental Sciences (Q1: 37/229)				
11	Journal of Water and Health	87 (0.73)	Environmental Sciences (Q4: 183/229)	1.041 (17)	14 (13)	16.09 (15)	England (UK)
			Microbiology (Q4: 109/124)				
12	Waste Management & Research	85 (0.71)	Engineering, Environmental (Q3: 28/49)	1.803 (13)	14 (13)	16.47 (14)	England (UK)
			Environmental Sciences (Q3: 117/229)				
13	Plos Neglected Tropical Diseases	79 (0.66)	Parasitology (Q1: 6/36)	3.834 (6)	20 (6)	25.32 (7)	USA
			Tropical medicine (Q1: 1/19)				
14	Environmental Science & Technology	77 (0.64)	Engineering, Environmental (Q1: 4/49)	6.198 (2)	27 (3)	35.06 (1)	USA
			Environmental Sciences (Q1: 12/229)				
15	Environment and urbanization	74 (0.62)	Environmental Studies (Q2: 41/105)	1.986 (12)	19 (8)	25.68 (6)	England (UK)
			Urban Studies (Q1: 9/38)				
16	Science of The Total Environment	72 (0.60)	Environmental Sciences (Q1: 22/229)	4.900 (3)	20 (6)	27.78 (4)	Netherlands
17	Environmental Monitoring and Assessment	72 (0.60)	Environmental Sciences (Q3: 126/229)	1.687 (14)	14 (13)	19.44 (11)	Netherlands
18	Tropical Medicine & International Health	63 (0.53)	Public, environmental & occupational health (Q1: 39/176)	2.850 (8)	19 (8)	30.16 (3)	England (UK)
			Tropical medicine (Q1: 2/19)				
19	Engenharia Sanitaria E Ambiental	60 (0.50)	Water Resources (Q4: 85/88)	0.222 (20)	3 (20)	5.00 (20)	Brazil
20	Journal of Environmental Management	58 (0.49)	Environmental Sciences (Q1: 39/229)	4.010 (5)	13 (16)	22.41 (9)	England (UK)

Note: TA: quantities of articles; JIF: journal impact factor; R; ranking in top20 productive journals; *: the category here belonged to SSCI not SCI-EXPANDED.

**Fig. 4:**
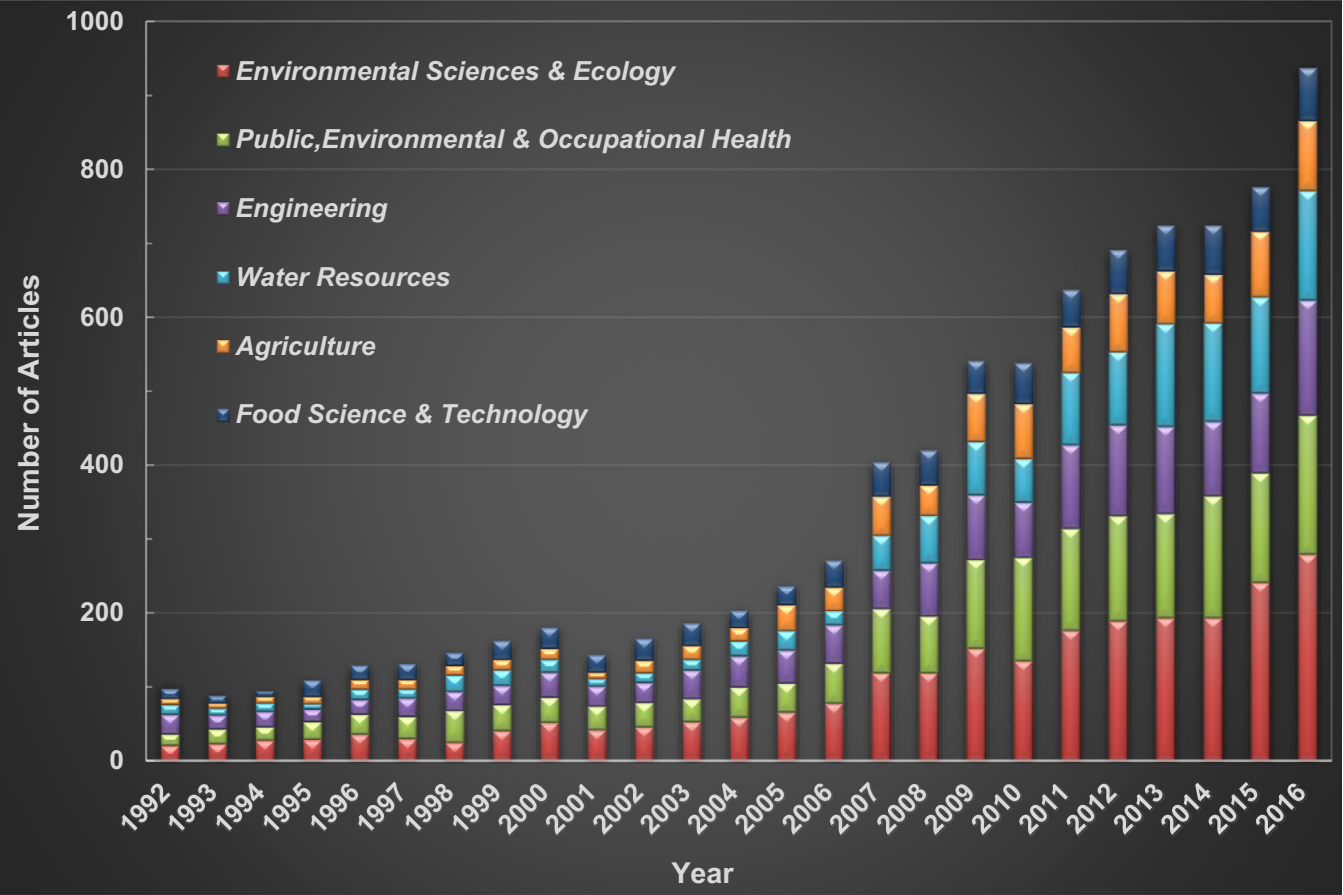
Growth tendency of the 6 most productive subject categories (TA > 900).

Since journals could be assigned to multiple subject categories in this research, sanitation research actually encompassed 143 subject categories. Fig. 4 shows that, based on the continuous increase in the number of articles per category, sanitation research mirrored the types of patterns found in other fields at the start of the new millennium. The most common subject categories were “Environmental Sciences & Ecology”, “Public, Environmental, & Occupational Health”, “Engineering”, and “Water Resources”, followed distantly by the second echelon of categories, which occupied a percentage between 10 and 15%. “Agriculture” and “Food Science & Technology” dominated the third level. The six most common subject categories were about the environment, water, health, and food, which may be due to the fact that sanitation is always associated with water and hygiene. In addition, an increased awareness of the nutrients contained in human excreta and the recovery of those nutrients for farming are gaining increasing attention. Such research is related to agriculture and food security (Lienert et al., 2003).

#### 3.1.3 Publication performance of countries and institutes

**Fig. 5:**
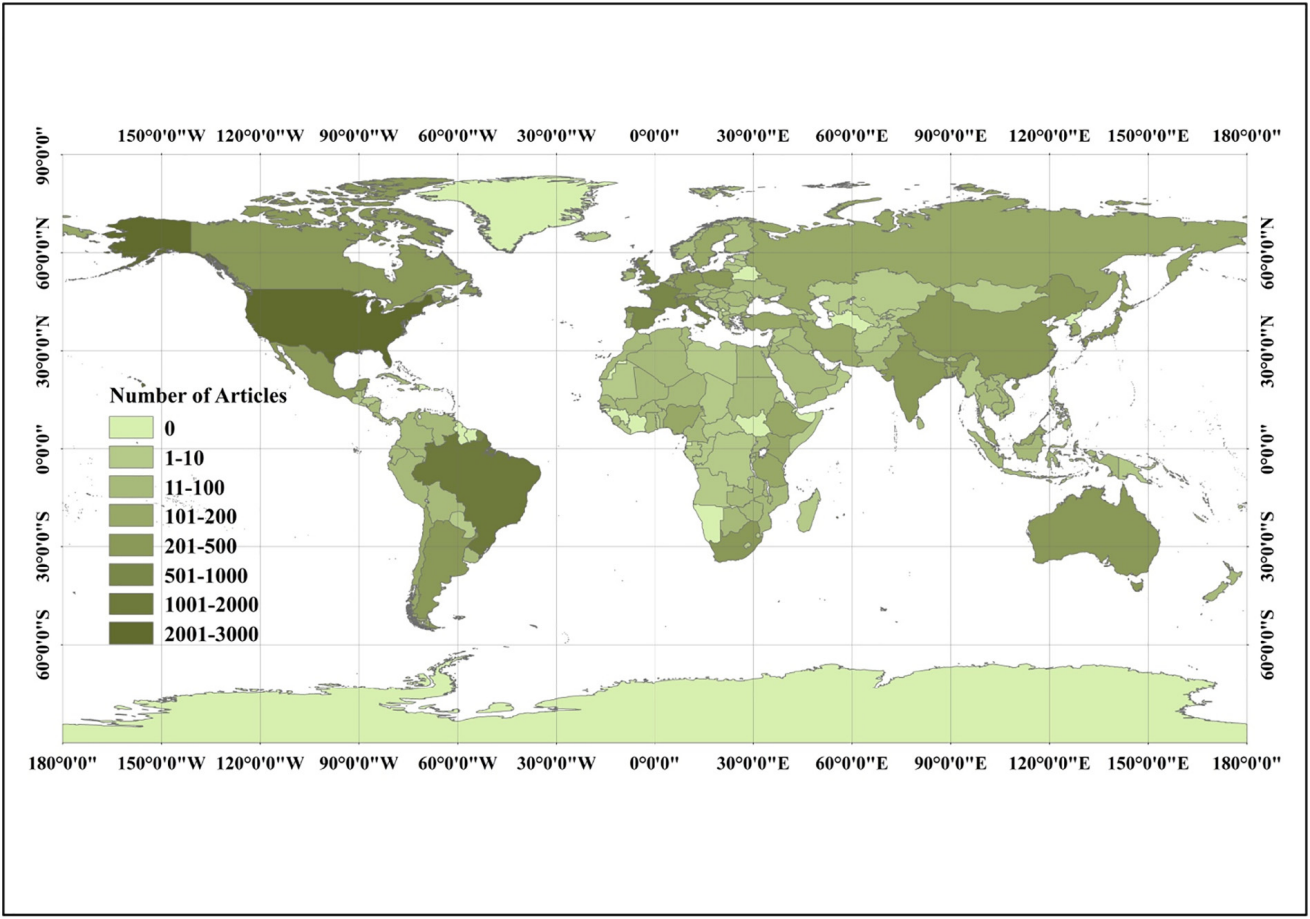
Global geographic distribution of authors according to the total number of articles by country/region.

By using at least one author's affiliation, we were able to identify which countries and areas were the most influential in the publication of sanitation research. We analyzed 11,956 articles with the author address information published between 1992 and 2016, which included 11,781 institutes in 182 countries/regions. There were 8978 (76.2%) independent articles and 2803 (23.8%) internationally-collaborative articles. Our analysis addressed the 20 most prolific countries. We examined the total number of works from a single country as well as internationally-collaborative research. Table 3 showed our data. This table also included the percentage of single country and internationally collaborative articles and the *h*-index for each area. The top 20 countries included three Asian countries/regions, ten European countries, five American countries, one Oceanic country, and one South African country. The 20 most productive countries generated the vast majority of published articles (94.5%). Almost four-fifths (78.6%) of the sanitation articles examined in this analysis were from Canada, France, Germany, Italy, Japan, the UK, and the USA. These developed countries have superior economic capabilities and higher academic levels than those of developing nations (Bell and Pavitt, 1993). Of the 182 regions we examined, Brazil came in second, India was seventh, China was ranked tenth, and South Africa was a distant 13th (Finardi, 2015). Russia was not included in the top 20. Fig. 5 shows the global geographic distribution of authors according to the total number of articles by country/region.

**Table 3:** The performance of the top 20 most productive countries (1992–2016).

Country/territories	TA	R (%)	Single country		Internationally-collaborated			*h*-index [R]	*h*-index/TA % [R]
			SA	%	CA	% [R]	MC [A]		
USA	2680	1 (22.7)	1648	61.49	1032	38.51 (10)	UK (187)	87 (1)	3.25 (19)
Brazil	1543	2 (13.1)	1319	85.48	224	14.52 (18)	USA (63)	40 (5)	2.59 (20)
UK	934	3 (7.9)	332	35.55	602	64.45 (4)	USA (187)	53 (2)	5.67 (17)
France	698	4 (5.9)	447	64.04	251	35.96 (13)	USA (41)	42 (4)	6.02 (16)
Spain	698	4 (5.9)	505	72.35	193	27.65 (16)	USA (27)	38 (6)	5.44 (18)
Italy	557	6 (4.7)	402	72.17	155	27.83 (15)	USA (42)	37 (7)	6.64 (15)
India	436	7 (3.7)	276	63.30	160	36.70 (11)	USA (66)	34 (8)	7.80 (12)
Switzerland	396	8 (3.4)	98	24.75	298	75.25 (1)	USA (95)	46 (3)	11.62 (3)
Germany	390	9 (3.3)	216	55.38	174	44.62 (7)	USA (31)	34 (9)	8.72 (8)
China	384	10 (3.3)	231	60.16	153	39.84 (9)	USA (54)	29 (12)	7.55 (13)
Canada	374	11 (3.2)	185	49.47	189	50.53 (6)	USA (69)	32 (10)	8.56 (10)
Japan	312	12 (2.6)	199	63.78	113	36.22 (12)	USA (20)	27 (14)	8.65 (9)
South Africa	284	13 (2.4)	159	55.99	125	44.01 (8)	USA (43)	24 (17)	8.45 (11)
Netherlands	273	14 (2.3)	86	31.50	187	68.50 (2)	UK (32)	27 (15)	9.89 (5)
Australia	271	15 (2.3)	87	32.10	184	67.90 (3)	USA (51)	30 (11)	11.07 (4)
Mexico	250	16 (2.1)	168	67.20	82	32.80 (14)	USA (34)	22 (18)	8.80 (7)
Poland	234	17 (2.0)	211	90.17	23	9.83 (20)	France (6)	17 (20)	7.26 (14)
Argentina	224	18 (1.9)	175	78.13	49	21.88 (17)	USA (17)	21 (19)	9.38 (6)
Sweden	191	19 (1.6)	68	35.60	123	64.40 (5)	UK (24)	28 (13)	14.66 (1)
Turkey	171	20 (1.5)	149	87.13	22	12.87 (19)	USA (9)	25 (16)	14.62 (2)

Note: TA: quantities of articles, R (%): rankings in TA, SA: Single-country article, CA: internationally-collaborated article, MC [P]: major collaborator.

**Fig. 6:**
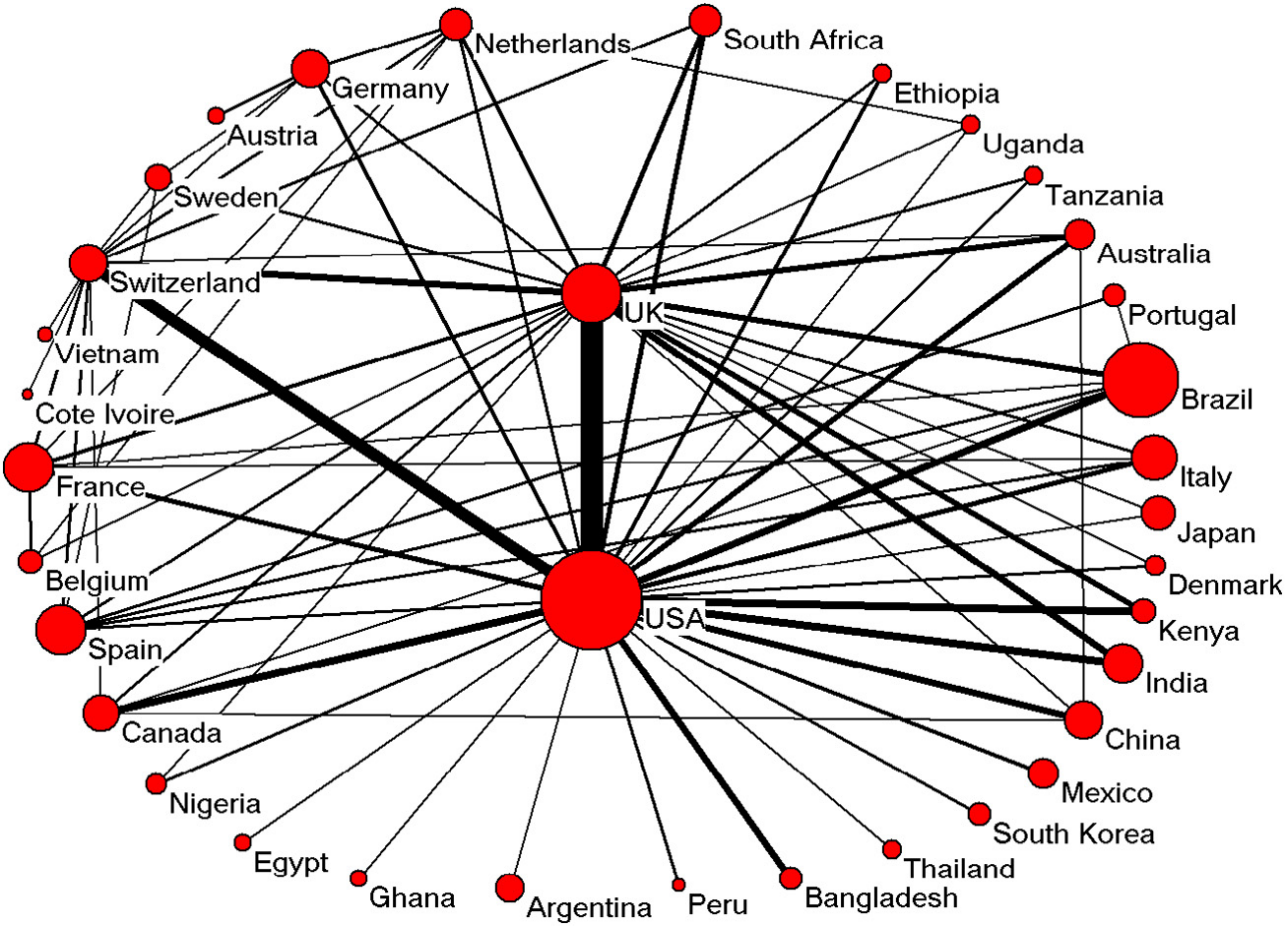
The cooperation network diagram among countries with no fewer than 17 articles.

The USA appeared at or near the top in our results, indicating that it is a global leader in sanitation studies and publications. The USA had the highest number of total articles published (2680). It was also first for its independent articles (1648) and first authored articles (2195). The USA had an *h*-index of 87 and published 1032 corresponding author articles. Sixteen nations partnered with researchers from the USA, and these collaborator data are shown in Table 3. Switzerland, Netherlands, and Australia are the top 3 collaborative countries. The total number of articles from China ranked 10th, and the percentages for independent and collaborated articles were 60.2% and 39.8%, respectively. We examined 35 countries that had at least 17 publications in order to highlight the trends in international cooperation. First, we placed the countries into clusters. We then used BibExcel and an algorithm from Persson (1994) and graphed our results with Pajek. Fig. 6 shows the results of our analysis. Each country (shown as a circle) is sized according to the weight of its publications. The line weight of each circle identifies the nation's research cooperation with authors from other regions.

The most productive countries are the USA and UK. They both have outsized and complex groups; 28 belong to the USA cluster and 22 groups belong to the UK cluster. Switzerland, France, and Belgium come next, but they have significantly lower numbers of articles and cooperation. The USA-UK collaborations ranked first, with 187 cooperative articles, followed by USA-Switzerland (95), USA-Canada (69), USA-India (66), USA-Brazil (63), and China-USA (54) collaborations. These articles covered several aspects, including social, political, environmental, public health, technical aspects and so on, which were mainly related to the context of developing countries. The descriptions of these articles expressed three phases for sanitation development, the first phase is the baseline study, to investigate the main sanitation related issues, e.g. technologies for disinfection and hygiene, which could be effective to prevent the transmission of disease; the second phase is the realization of MDG, global estimation of the indicators according to MDGs; the third phase is the development of new technology for sanitation with the concept of closing the loop in order to reach the SDGs.

For the techniques developed by the USA, during the MDGs period, the target was to get improved sanitation, intended for safe separation of humans from excreta by employing flushing toilets, septic tanks, latrines, composting toilets etc., but not a goal of hygienization or source recovery. Thus, the main technologies were composting and storage processes. Recently, because of the implementation of SDGs and encouragement by the Bill and Melinda Gates Foundation, some non-sewer and source oriented toilet systems have been developed, as mentioned in the Reinvent the Toilet Challenge manuscript. From the year 2011 to 2013, 15 Grants have been awarded for innovated toilet developers, and 6 of them are from USA, further supporting the statements. Table 4 below lists brief descriptions of the 6 reinvented toilet technologies developed by USA.

**Table 4:** Brief information of the 6 reinvented toilet technologies developed by USA.

Description of the technology	Authorship
To develop a self-contained, solar-powered toilet and wastewater treatment system. A solar panel will produce enough power for an electrochemical reactor that is designed to break down water and human waste. Excess power can be stored to provide energy for nighttime operation or for use under low-sunlight conditions.	California Institute of Technology, USA
To develop a self-contained system that pyrolyzes (decomposes at high temperatures without oxygen) human waste into biochar. Energy recovered from the biochar production process will be used for heating the system.	Stanford University and the Climate Foundation, USA
To develop a self-contained toilet system that disinfects liquid waste and turns solid waste into fuel or electricity through a novel biomass energy conversion unit.	RTI International, USA
To develop a solar toilet that uses concentrated sunlight, directed and focused with a solar dish and concentrator, to disinfect liquid-solid waste and produce biochar that can be used as a replacement for wood charcoal or chemical fertilizers.	University of Colorado Boulder, USA
To develop, build, and evaluate a novel technique to treat fecal sludge using supercritical water oxidation, a process in which water is heated under pressure and then oxygen is added to burn up human waste. The reaction produces clean water, heat, carbon dioxide, benign salts, and nitrogen, all of which can be used by the community or turned into business opportunities.	Duke University, USA
To develop an electric toilet, powered by solar power stored in batteries, that will separate liquids from solids and dewater and convert fecal matter into biochar. This approach examines using resistive heating through battery-stored solar power and is designed from existing off-the-shelf components.	Santec LLC, USA

**Fig. 7:**
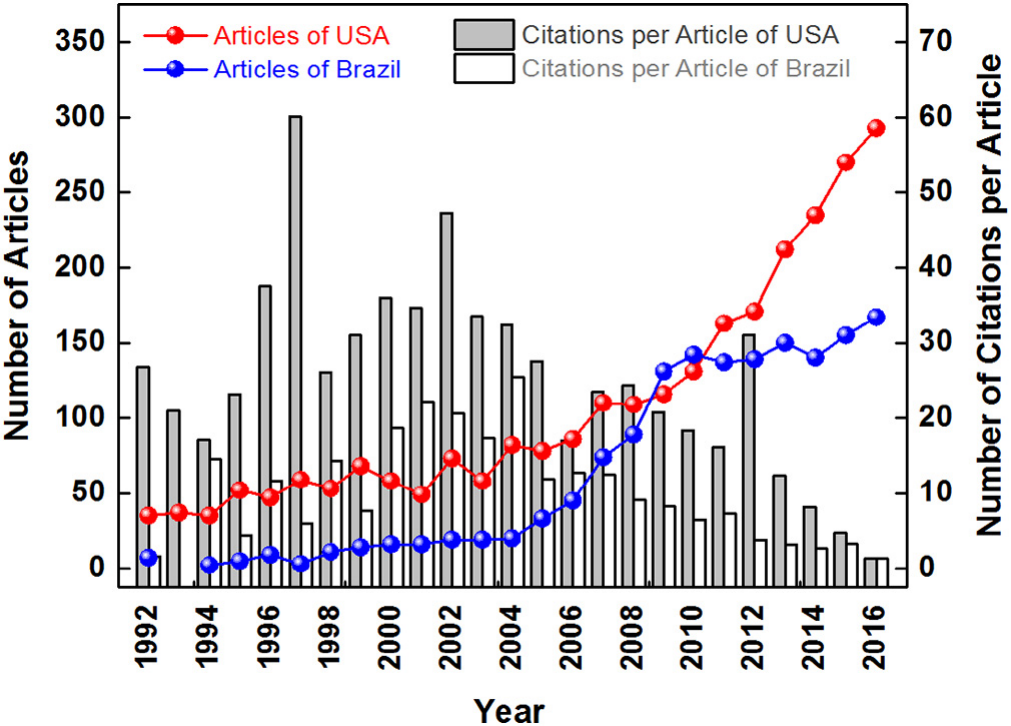
Trends in articles and citations from the USA and Brazil from 1992 to 2016 (TA > 1500).

Fig. 7 shows the trends and times cited per year for articles from the USA and Brazil. These nations were quite productive and had a similar history of research. The USA consistently published a high number of articles per year every year up until 2008. Then, in 2009 and 2010, Brazil was ranked first for number of articles. This trend did not continue, however, as in 2011, the USA moved back into the top slot.

Of the 11,956 articles spanning 11,781 institutes in 182 countries, 6890 (58.5%) were inter-institutionally collaborative publications and 4891 (41.5%) were independent publications. The collaboration between institutes was 34.7% higher than that between countries. The top 20 research facilities included 9 in the USA, 5 in Brazil, 3 in Switzerland, 2 in the UK, and 1 each in France and Spain; 8 of the 9 institutes in the USA were universities. These data are shown in Table 5.

**Table 5:** The performance of the top 20 most productive institutions (1992–2016).

Institution	TA	R (%)	Single-institution		Inter-institution collaborative			*h*-index [R]	*h*-index/TA % [R]
			SP	%	CP	%	MC(P)		
Univ Sao Paulo, Brazil	227	1 (1.9)	39	17.18	188	82.82 (11)	Univ Estadual Campinas, Brazil (17)	22 (5)	9.69 (21)
London Sch Hyg & Trop Med, UK	129	2 (1.1)	6	4.65	123	95.35 (4)	Emory Univ, USA (31)	22 (5)	17.05 (19)
Ctr Dis Control & Prevent, USA	115	3 (1.0)	15	13.04	100	86.96 (8)	Emory Univ, USA (9)	34 (1)	29.57 (7)
Emory Univ, USA	115	3 (1.0)	6	5.22	109	94.78 (5)	London Sch Hyg & Trop Med, UK (31)	20 (10)	17.39 (18)
Univ Fed Minas Gerais, Brazil	92	5 (0.8)	20	21.74	72	78.26 (17)	Fiocruz MS, Brazil; Univ Sao Paulo, Brazil (6)	11 (20)	11.96 (20)
Univ N Carolina, USA	88	6 (0.7)	17	19.32	71	80.68 (15)	Columbia Univ, USA (7)	27 (3)	30.68 (4)
Harvard Univ, USA	83	7 (0.7)	7	8.43	76	91.57 (7)	Univ Washington, USA (8)	23 (4)	27.71 (9)
Fundacao Oswaldo Cruz, Brazil	81	8 (0.7)	27	33.33	54	66.67 (20)	Univ Fed Rio de Janeiro, Brazil (13)	15 (19)	18.52 (16)
USDA ARS, USA	80	9 (0.7)	29	36.25	51	63.75 (21)	Univ Georgia, USA (9)	19 (13)	23.75 (12)
Univ Calif Davis, USA	78	10 (0.7)	17	21.79	61	78.21 (18)	Emory Univ, USA; London Sch Hyg & Trop Med, UK (9)	18 (15)	23.08 (13)
Univ Fed Bahia, Brazil	78	10 (0.7)	15	19.23	63	80.77 (14)	London Sch Hyg & Trop Med, UK (13)	18 (15)	23.08 (13)
Univ London London Sch Hyg & Trop Med, UK	76	12 (0.6)	15	19.74	61	80.26 (16)	Emory Univ, USA (12)	21 (8)	27.63 (10)
INRA, France	74	13 (0.6)	14	18.92	60	81.08 (13)	Agrocampus Ouest, France; Inst Elevage, France (5)	19 (13)	25.68 (11)
Univ Florida, USA	70	14 (0.6)	13	18.57	57	81.43 (12)	Emory Univ, USA (19)	16 (18)	22.86 (15)
Swiss Trop & Publ Hlth Inst, Switzerland	69	15 (0.6)	0	0.00	69	100.0 (1)	Univ Basel, Switzerland (61)	21 (8)	30.43 (5)
WHO, Switzerland	68	16 (0.6)	11	16.18	57	83.82 (9)	Ctr Dis Control & Prevent, USA (8)	28 (2)	41.18 (1)
Univ Basel, Switzerland	67	17 (0.6)	1	1.49	66	98.51 (2)	Swiss Trop & Publ Hlth Inst, Switzerland (61)	20 (10)	29.85 (6)
Univ Calif Berkeley, USA	61	18 (0.5)	5	8.20	56	91.8 (6)	Stanford Univ, USA; Emory Univ, USA (9)	20 (10)	32.79 (3)
Univ Fed Rio de Janeiro, Brazil	60	19 (0.5)	10	16.67	50	83.33 (10)	Fundacao Oswaldo Cruz, Brazil (13)	11 (20)	18.33 (17)
Johns Hopkins Univ, USA	60	19 (0.5)	2	3.33	58	96.67 (3)	Johns Hopkins Bloomberg Sch Publ Hlth, USA (8)	22 (5)	36.67 (2)
CSIC, Spain	60	19 (0.5)	15	25.00	45	75.00 (19)	Univ Seville, Spain (5)	17 (17)	28.33 (8)

Note: TA: quantities of articles, R (%): ranking in TA, SP: Single-institution article, CP: Inter-institution collaborative article, MC [P]: major collaborator.

The ‘*Center for Disease Control and Prevention*, U.S. Department of Health & Human Services’, ‘*WHO*’ and the ‘*University of North Carolina*’ are the top three institutions in h-index with high values of 34, 28, and 27, respectively, which further emphasizes the importance of sanitation for hygiene and disease control. Over half of the 20 institutes were universities, demonstrating that the universities are the core drivers of sanitation research.

### 3.2 Research trends and hotspots

#### 3.2.1 Keywords

**Fig. 8:**
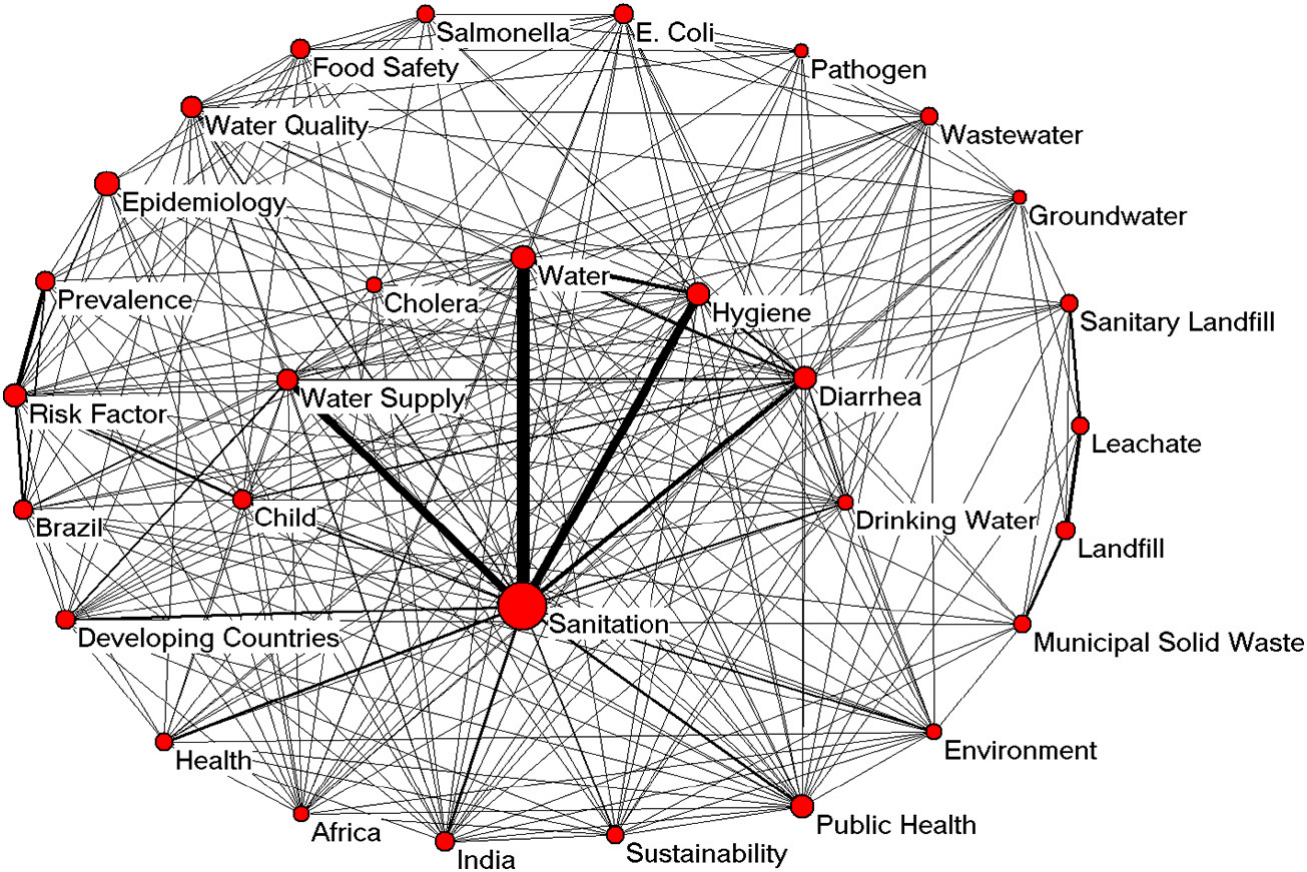
Network diagram of the top 30 author keywords from 1992 to 2016.

The statistical analysis of the author keywords, article title, article abstract, and “Keywords Plus” could be used to identify directions in science; keyword analysis is efficient for comprehending the progress of new frontiers in science (Li et al., 2011; Tan et al., 2014). The analysis of author keywords across different periods is a common bibliometric technique for analyzing research trends (Fu et al., 2014; Wang et al., 2015; Zhang et al., 2014; Zhang et al., 2010; Zheng et al., 2017). We excluded the 6493 articles without author keyword information and analyzed 11,956 articles with the author keyword information. There were 22,391 keywords listed by authors; however, 17,310 (77.3%) of the keywords were used only once and 2335 (10.0%) of the keywords were used twice. There were many author keywords that were only used one time. This single use points to the utilization of comprehensive subject keywords. It also indicates that there were differences in how researchers approached their work (Hou et al., 2015; Li and Zhao, 2015). The bulk of the sanitation research relied on a small number of keywords. For example, 511 (2.3%) keywords appeared >10 times. Table 6 shows the 30 most commonly used keywords. The changes in the rankings of words over the five-year intervals show that there were variations in the research hotspots. Fig. 8 shows the co-occurrence relationships of the 30 most commonly-used author keywords. Here, we created a co-word network to better illustrate the relationships between these terms.

**Table 6:** Top 30 utilized author keywords in 5 five-year periods (1992–2016).

Author keywords	92–16 TA	92–16 R (%)	92–96 R (%)	97–01 R (%)	02–06 R (%)	07–11 R (%)	12–16 R (%)
Sanitation	751	1 (8.3)	1 (8.8)	1 (5.7)	1 (8.6)	1 (6.7)	1 (9.6)
Epidemiology	205	2 (2.3)	2 (5.4)	2 (3.5)	4 (2.3)	3 (2.1)	4 (1.9)
Water	198	3 (2.2)	65 (0.6)	25 (0.9)	9 (1.7)	9 (1.6)	2 (3.0)
Public health	176	4 (1.9)	8 (2.0)	3 (2.2)	13 (1.4)	2 (2.2)	5 (1.9)
Hygiene	167	5 (1.8)	8 (2.0)	5 (2.0)	10 (1.6)	11 (1.5)	3 (2.1)
Diarrhea	160	6 (1.8)	4 (4.0)	11 (1.8)	6 (2.0)	7 (1.7)	9 (1.6)
Risk factor	159	7 (1.8)	19 (1.1)	5 (2.0)	13 (1.4)	4 (1.9)	6 (1.8)
Water supply	150	8 (1.7)	5 (3.4)	53 (0.6)	2 (2.9)	10 (1.5)	14 (1.4)
Water quality	149	9 (1.6)	10 (1.7)	8 (1.9)	13 (1.4)	11 (1.5)	6 (1.8)
Brazil	132	10 (1.5)	65 (0.6)	25 (0.9)	19 (1.2)	5 (1.8)	11 (1.5)
Prevalence	131	11 (1.4)	174 (0.3)	8 (1.9)	10 (1.6)	13 (1.4)	11 (1.5)
*E. coli*	130	12 (1.4)	65 (0.6)	25 (0.9)	5 (2.2)	15 (1.3)	10 (1.5)
Food safety	126	13 (1.4)	*N/A*	19 (1.2)	27 (1.0)	8 (1.7)	11 (1.5)
Landfill	121	14 (1.3)	6 (2.5)	12 (1.6)	3 (2.4)	17 (1.1)	21 (1.0)
Developing countries	120	15 (1.3)	15 (1.4)	22 (1.0)	7 (1.8)	6 (1.7)	24 (1.0)
India	115	16 (1.3)	65 (0.6)	158 (0.3)	27 (1.0)	16 (1.2)	8 (1.6)
Child	111	17 (1.2)	65 (0.6)	15 (1.3)	12 (1.5)	18 (1.1)	15 (1.3)
Sanitary landfill	101	18 (1.1)	3 (4.2)	5 (2.0)	7 (1.8)	31 (0.7)	31 (0.8)
Wastewater	97	19 (1.1)	31 (0.8)	158 (0.3)	38 (0.7)	14 (1.3)	19 (1.2)
Health	95	20 (1.1)	174 (0.3)	34 (0.7)	32 (0.8)	20 (1.0)	17 (1.2)
Salmonella	93	21 (1.0)	15 (1.4)	19 (1.2)	23 (1.1)	24 (1.0)	22 (1.0)
Municipal solid waste	93	21 (1.0)	65 (0.6)	15 (1.3)	16 (1.3)	25 (0.9)	22 (1.0)
Sustainability	92	23 (1.0)	*N/A*	158 (0.3)	38 (0.7)	19 (1.1)	15 (1.3)
Leachate	92	23 (1.0)	7 (2.3)	8 (1.9)	19 (1.2)	20 (1.0)	41 (0.7)
Drinking water	90	25 (1.0)	174 (0.3)	53 (0.6)	50 (0.6)	23 (1.0)	18 (1.2)
Environment	78	26 (0.9)	19 (1.1)	13 (1.5)	38 (0.7)	25 (0.9)	35 (0.7)
Africa	76	27 (0.8)	174 (0.3)	15 (1.3)	50 (0.6)	27 (0.9)	27 (0.8)
Cholera	74	28 (0.8)	174 (0.3)	53 (0.6)	60 (0.5)	43 (0.6)	20 (1.1)
Groundwater	71	29 (0.8)	19 (1.1)	25 (0.9)	32 (0.8)	50 (0.6)	25 (0.9)
Pathogen	66	30 (0.7)	10 (1.7)	34 (0.7)	27 (1.0)	38 (0.6)	46 (0.6)

Note: TA: quantities of articles, R (%): ranking and percentage in author keywords, and *NA*: not available.

Because the terms “sanitation” and “sanitary” were the subject words in this study, the most frequently-used keyword (“sanitation”) was ignored. Table 5 and Fig. 8 show the outcomes of our keyword analysis. The three most frequently-used keywords were “epidemiology” (205; 2.3%), “water” (198; 2.2%), and “public health” (176; 1.9%). These top three most frequently-used substantives reflect the large amount of literature dealing with water and hygiene, especially the diseases transferred with poor sanitation, such as diarrhea, lower respiratory illnesses, and other common infectious diseases (Lim et al., 2012). Fig. 8 shows that “water” is closely related to “sanitation”. This keyword was increasingly studied and enjoyed a rise in rank from 65th in 1992–1996 to 2nd in 2012–2016. This increase showed a growing awareness of the importance of proper and efficient water management for sanitation improvement, especially a safe water supply and source-oriented wastewater treatment. “Water supply” (150, 1.7%), “water quality” (149, 1.6%), “wastewater” (97, 1.1%), and “drinking water” (90, 1.0%) ranked 8th, 9th, 19th, and 25th, respectively, as the most frequently-used keywords. Besides “hygiene”, “diarrhea”, and “risk factor”, “Brazil” was one of the top 10 keywords during all 5 five-year periods, which illustrates that water, sanitation, and hygiene are on-going concerns. The pathogenic indicator bacteria “*E. coli*” and “*Salmonella*” ranked 12th and 21st, respectively, in the most frequently used keywords, which indicate that these bacterial species have been extensively used as indicator pathogens for hygienization. *E. coli* had an apparent upward movement in the ranks, from 65th in 1992–1996 to 10th in 2012–2016, which could be attributed to the fact that *E. coli* is commonly observed in the gut of humans and warm-blooded animals. This trend also reflects the awareness of the public's health. The names of developing countries (120, 1.3%) ranked 15th in the top 30 most frequently-used substantives in author keywords; they were initially ranked 15th in 1992–1996, then moved to 22nd in 1997–2001, 7th in 2002–2006, and finally to 6th place in 2007–2011. However, they decreased to 24th in 2012–2016. This trend could be explained by the continuing attention given to a specific area and country, especially low-income districts. Hence, “India” (115, 1.3%) and “Africa” (76, 0.8%) ranked 16th and 27th, respectively, in the top 30 most frequently-used keywords, and both of these keywords had a significant increase over the past 25 years. “India” and “Africa” ranked 158th and 174th, respectively, in 1997–2001 and 8th and 27th, respectively, in 2012–2016. “Child” (111, 1.2%) ranked 17th in the top 30 keywords, but it was ranked 65th from 1992 to 1996 and then it moved to 10th place in 2012–2016. This trend occurred because children are the most sensitive to poor sanitation. “Sustainability” has become increasingly popular in recent years, moving up in the rankings from “not available” in 1992–1996 to 158th in 1997–2001, 38th in 2002–2006, 19th in 2007–2011, and 15th in 2012–2016. From 1992 to 1996, improved sanitation was a primary focus of research. It was important to ensure the hygienic separation of human excreta from human contact and the suggested facilities that could reach the targets included flush/pour-flush-to-piped-sewer systems or septic tanks/pit latrines, ventilated improved pit (VIP) latrines, pit latrines with slabs, and composting toilets. This explains why sustainability was barely mentioned during the first 5 years in our study. Since the awareness of protecting human health and the environment has increased, the concept of sustainable sanitation has been promoted, and sustainability has garnered increasing attention.

#### 3.2.2 Hotspots

**Fig. 9:**
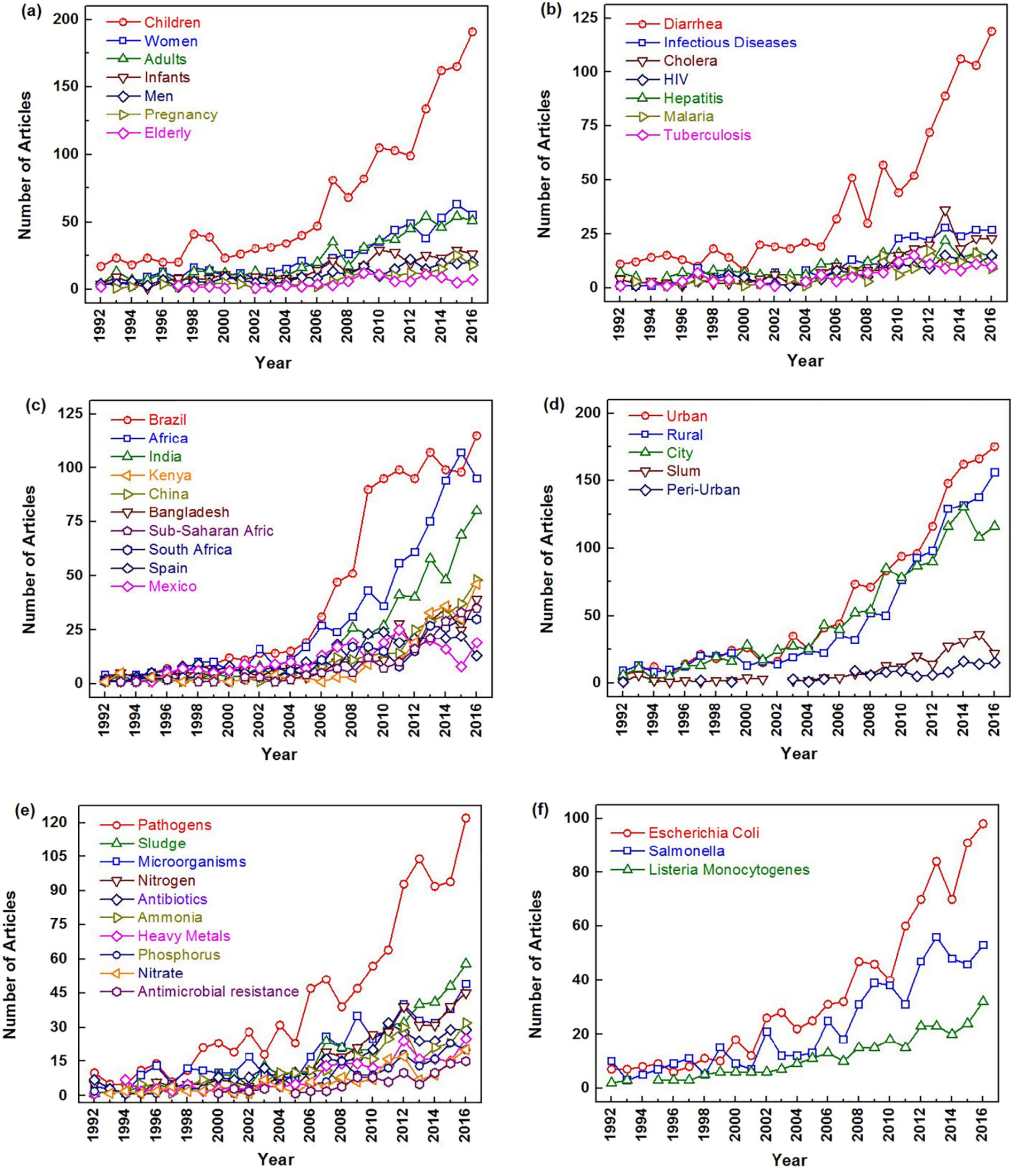
Growth tendency of hotspot articles from 1992 to 2016.

Baskerville (1904) showed that patterns and ideas in research can be identified via author keywords and the title of articles. We therefore used an algorithm to generate “KeyWords Plus”. These terms were drawn from the titles of works that appeared in the articles (Garfield, 1990).We also utilized a relatively new method in this analysis, Mao et al.'s “word cluster analysis” (Mao et al., 2010). The word cluster analysis uses the author keywords, article title, article abstracts, and KeyWords Plus to identify patterns in data. Many other researchers have used this approach to determine research hotspots (Fu et al., 2013; Qian et al., 2015; Tan et al., 2014). The possible research hotspots for sanitation research were divided into 6 categories, including “susceptible population”, “epidemic disease”, “specific countries”, “different regions”, “concerned contaminates”, and “characterized microorganisms”; all the categories were closely related to hygienization. Fig. 9 shows the growth trend of hotspot-related articles during the last 25 years in the six categories.

UNICEF noted that clean water, basic toilets, and good hygiene practices are essential for the survival and development of children. Fig. 9a and b show that children suffer primarily from diarrhea. The WHO website noted that every year, 361,000 children under five years of age die from diarrhea. The transmission of diseases is linked to poor sanitation and water contamination. One report titled “A fair chance for every child” released by UNICEF stressed the importance of sanitation to children's health and mortality; it noted that child mortality could be reduced by improved sanitation facilities (UNICEF, 2016). Hence, improving sanitation services and basic hygiene practices for children is a critical hotspot. Due to the limitations of their economies, developing countries face greater challenges in improving sanitation conditions; evidence for these challenges can be found in Fig. 8c, which shows the hotspots of research work in specific countries. Brazil, Africa, and India are the top three countries/regions according to the number of articles collected from the database (115,95, and 80, respectively). The rest of the top 10 countries/regions are Bangladesh, Spain, China, Mexico, South Africa, Kenya, and Sub-Saharan Africa. Most of these nations are defined as developing countries. The rapid increase in research in Brazil may be due to the rapid urbanization of Latin America in the last few decades. Even though there were 28.8 million people having access to “at least basic” sanitation, and 11% of the population used “unimproved” sanitation (pubic or shared latrine, open pit latrine, bucket latrine), 2% of the population still practiced open defecation as of 2015 (Watch, 2016). Most African countries, including South Africa, Sub-Saharan Africa, and Kenya, lacked clean water, basic sanitation, and good personal hygiene, which are the results of extreme poverty. In Burkina Faso, only 7% of the rural population had access to improved household sanitation (hygienically separates human excreta from human contact); 75% still defecated in the open in 2015 (UNICEF, 2015). Asian countries, such as India, Bangladesh, and China, show increasing improvement. India (with almost 62 million children, 48% of the population) has the largest number of the world's growth-stunted children in Asia, where the key issue is a lack of toilets rather than lack of food (Worley, 2014). Thus, improving the sanitation conditions in India has garnered attention from the government and academia. In 2013, the Department of Biotechnology (DBT), under auspices of the Ministry of Science and Technology of the Government of India, and the BMGF, in collaboration with India's Biotechnology Industry Research Assistance Council (BIRAC), launched “RTTC-India”. Six grantees were chosen from 108 applicants to develop innovative, affordable, and scalable sanitation technologies, with a total grant of $2 million (Mehta, 2014). In China, 98% of the urban population has access to improved toilets (as of 2012); the coverage of sanitary toilets in rural areas was 7.5% in 1993, 74.1% in 2013, and 78.4% in 2015, indicating that great headway was made in building sanitary toilets over the last 15 years (China, 2015; PRC, 2016). Similarly, the BMGF has been funding RTTC activity in China since 2013, which encouraged researchers from universities, research institutes, enterprises, and other social groups to develop a new generation of toilets and increased the number of academic publications on the subject (Liu et al., 2017; Wang et al., 2017). With the implementation of the nationwide “Toilet Revaluation”, greater achievements are expected during China's “13^th^ Five-year Plan”. The majority of those without improved sanitation are people who live in rural areas, thus, rural areas are characterized by the great disparity in sanitation improvements. However, Fig. 8d shows that “rural”, “city”, and “urban” appear frequently in research; the number of articles with “urban” even exceeded the number of articles that included “rural” in the keywords. The percentage of the urban population living in South Sudan without safe toilets accounted for 83.6% of the total, which dominates the list, while India has 157 million urbanites living without sanitation, ranking first for the most people without access to proper toilet facilities (WaterAid, 2016). The concept of sustainable sanitation not only concerns toilets, but also the proper disposal and reuse of excreta. Therefore, urban areas that are far away from the main sewers urgently require innovative sanitation techniques. An announcement made on the fifth World Toilet Day (the ISO 30500) titled it “Non-sewered sanitation systems – Prefabricated integrated treatment units – General safety and performance requirements for design and testing”) indicated that WHO would provide international standardized regulation for sanitation systems worldwide, including urban communities without access to sewer systems, and the organization would pursue sustainable sanitation solutions (Lazarte, 2017; Lewis, 2017).

With the transformation of the concept of sanitation from “improved sanitation” to “sustainable sanitation”, excreta and wastewater are not recognized as hazardous waste, but valuable resources that can be reused and recycled. Hence, a better understanding of the composition of waste is needed. Fig. 9e reveals that “pathogens”, “sludge”, “nitrogen”, “microorganisms”, “antibiotics”, “ammonia”, “phosphorus”, “nitrate”, “heavy metals”, and “antimicrobial resistance” were the top ten research hotspots in scientific research, and they are the main components of human excreta. These keywords were increasingly being used in research articles. Based on the characteristics of these pollutants, the research hotspots can be classified into three categories: (i) hygienization of excreta, (ii) resource (such as nitrogen, phosphorus, and electricity) recovery from excreta, and (iii) micro-pollutant control of excreta, such as antibiotics and heavy metals. Antimicrobial resistance is a new concern from a hygienization point of view due to the increasing amount of antibiotics in water systems.

(i) **Hygienization of excreta**: The hygienization of human excreta is essential for reducing the transmission of diseases by pathogenic microorganisms. Human urine contains very few, if any, pathogens, but the amount increases due to cross-contamination with feces. Nevertheless, the health risk can be eliminated through natural attenuation (Hoglund et al., 2000) or existing disinfection technologies. The behaviors of bacteria, protozoan pathogens, and viruses in stored-source-separated urine were studied, and it was demonstrated that the inactivation of pathogenic microorganisms depended on either the temperature and pH or only the temperature (Hoglund et al., 2002a, Hoglund et al., 2002b, Hoglund et al., 1998). For safe agricultural applications, WHO recommends a storage period of 6 months at 20 °C or higher. Stored urine could then be used to fertilize crops without restriction (World Health Organization, 2006). In order to increase the inactivation efficiency and reduce storage time, researchers increased the storage temperature. The inactivation of *E. coli*, Salmonella, and MS2 at these higher temperatures was faster than that which occurred in the urine stored at ambient temperature (Nordin et al., 2013; Vinneras et al., 2008; Zhou et al., 2017). The rapid inactivation of bacteria could be achieved by coupling other technologies with this process, such as acidification (Andreev et al., 2017)and nitrification (Bischel et al., 2015). Similarly, self-sanitization by ammonia and a high temperature is also effective for fecal sludge, but the time needed to eliminate pathogens is longer than that needed for urine due to the large amount and species of pathogens, viruses, and helminths in it (Fidjeland et al., 2015; Magri et al., 2015; Magri et al., 2013). Alkaline additives, such as ash, sawdust, and urea, could speed up the hygienization process (Fidjeland et al., 2013, Niwagaba et al., 2009a, Niwagaba et al., 2009b). Thermal treatments have been applied to fecal sludge treatment, and other treatment processes, like composting and anaerobic digestion, could have an effect on pathogen inactivation at some level (Vinneras, 2007; Yin et al., 2018). Fig. 8f shows that *E. coli* is the primary indicator microorganism in scientific research; it is also recommended as an indicator of health risk from water contact by the US Environmental Protection Agency (US EPA). It is followed by *Salmonella* and *Listeria monocytogenes*.

(ii) **Resource recovery from excreta**: Urine accounts for only ∼1% of the total volume of domestic wastewater, but it contains >80% of the nitrogen (N) and 50% of the phosphorus (P) load in domestic wastewater (Larsen and Gujer, 1996). Thus, the recovery of nutrients is much more strongly promoted than their removal, especially in light of the phosphorus crisis (Gilbert, 2009). The average amounts of phosphate, nitrogen, and potassium in human urine are approximately 5.6 kg, 0.5 kg, and 1.0 kg per person per year, accounting for 37%, 19%, and 54% of the global consumption of fertilizer. The main form of nitrogen in urine is ammonia due to the hydrolysis of urea during storage, while the phosphorus in urine exists as inorganic phosphate ions. Feces contain some nutrients and organic material, which can act as good soil conditioners after aerobic stabilization. Generally, the most direct way to use urine and feces as fertilizer for agriculture occurs after proper processing, especially in low-income areas or regions dominated by agriculture. For urine, the suggested approach for its safe reuse is long-term storage, and the storage time is dependent on the temperature. As noted earlier, WHO recommends 6 months of storage before urine can be safely used for crops. The most direct approach for using feces as a fertilizer or soil conditioner is to utilize it after composting. Composting is also effective for sanitizing fecal sludge. Composting is a self-heating microbial process, and it is effective for pathogenic bacteria inactivation at temperatures over 50 °C for a specific time period (Niwagaba et al., 2009a, Niwagaba et al., 2009b). Much research has been conducted to investigate the composting process, including co-composting with other carbon-rich materials, like kitchen waste, wood chips, and saw dust (Mahmood et al., 2015; Mulec et al., 2016; Sossou et al., 2014). Fecal sludge recycling after anaerobic digestion is another alternative that provides fertilizer as well as energy recovery (biogas). The reuse of urine and feces after stabilization and hygienization by storage or composting/digestion has been in practice for many years since it is simple and easy to manage. However, the loss of nutrients and secondary pollution that occur during the transportation of liquids is a concern. Therefore, newly-developed engineering technologies have become viable alternatives for the efficient commercial recovery of nutrients.

Ammonia stripping and struvite precipitation are two of the most commonly employed techniques in nutrient recovery from source-separated urine. Ammonia stripping is a physicochemical process that strips ammonium to gaseous NH_3_, which is then recovered as liquid ammonia, ammonium sulfate, or ammonium carbonate (Maurer et al., 2006; Nancharaiah et al., 2016). >90% of the nitrogen could be recovered by stripping under optimized operating conditions (Antonini et al., 2011, Basakcilardan-Kabakci et al., 2007). Kinetic analysis showed that higher air flow rates and temperatures could improve the recovery efficiency so as to decrease the unit operating cost. An extremely high pH is not recommended for the operation, even though it could increase the efficiency, because it may result in increased costs (Liu et al., 2015). Stripping only recovers nitrogen, while struvite recovers both nitrogen and phosphorus. Struvite is magnesium ammonium phosphate (MgNH_4_PO_4_·6H_2_O, MAP), which is the chemical precipitation that occurs at an alkaline pH with a suitable ratio of ammonium, phosphorus, and magnesium. This process converts nutrients from liquid to solid form, and its products can be used as a slow-release fertilizer (Maurer et al., 2006). The addition of magnesium is necessary for struvite formation to meet the chemical equilibrium of the constituent ions in the solution. Major parameters, such as the magnesium dosage, mixing rate, and pH, have been extensively investigated at lab scale (Ronteltap et al., 2007; Tilley et al., 2008; Wilsenach et al., 2007). It was reported that 90% of the phosphorous and approximately 20% of the nitrogen could be recovered through MAP crystallization. A combination of ammonia stripping and struvite precipitation to improve nitrogen and phosphorus recovery has been investigated. One investigation employed Ca (OH)_2_ to replace NaOH to make the stripping and precipitation occur at the same time; this study showed that 85–99% of the nitrogen and 99% of the phosphorus (w/w) can be harvested from hydrolyzed urine in 28 h at 40 °C and in 32 h at 30 °C (Pradhan et al., 2017). Although 90% of the phosphorus and approximately 20% of the nitrogen could be recovered through MAP crystallization, nearly all of the potassium would be lost, so magnesium potassium phosphate hexahydrate (MgKPO_4_·6H_2_O, MPP) crystallization was developed to simultaneously recover phosphorus as well as potassium (Xu et al., 2012; Xu et al., 2015; Zhang et al., 2017). The ferric ion (Fe^3+^) has also been used for phosphorus recovery (Jadhav and Hocheng, 2016). It was estimated that the available phosphorus from urine and feces produced in urban settings is approximately 0.88 million metric tons and will increase with population growth to over 1.5 million metric tons by 2050 (Mihelcic et al., 2011). Thus, recovering phosphorus from urine and feces will continue to be an attractive option. Other technologies that transform the wastewater treatment system, such as ion exchange, membranes, and bio-electrochemical systems (BES”s), have also been investigated. BESs were a critical research hotspot recently because they provide for on-site treatment, broad contamination removal, and energy production; they are also easy to manage and operate (Zollig et al., 2017), since they use microorganisms to catalyze oxidation and/or reduction reactions at the electrodes (e. g., the anode or cathode) collectively (Nancharaiah et al., 2016). Microbial Fuel Cells (MFCs) and Microbial Electrolysis Cells (MECs) are two key microbial electrochemical technologies (Ledezma et al., 2015). It was first demonstrated in 2012 that MFCs generated electricity from diluted urine, and this electricity could be used to power cell phones (Ieropoulos et al., 2013). Subsequently, research on enhanced MFC systems aims to improve the cathode structure, provide for more efficient nutrients/energy recovery, and support pathogen inactivation (Ieropoulos et al., 2017, Merino-Jimenez et al., 2017, Salar-Garcia et al., 2017, Zang et al., 2012). MECs are normally associated with hydrogen production and nutrient recovery (Kuntke et al., 2014), and researchers are focusing on efficient nutrient recovery by controlling the load ratio, changing the electric current conditions, and developing electrodes (Dbira et al., 2015; Rodriguez Arredondo et al., 2017; Yuan and Kim, 2017). To date, many technologies have been developed and modified, which demonstrates the need for innovation and specialization to meet the different requirements for various countries and regions.

(iii) **Micro-pollutant control of excreta**: Sustainable sanitation takes into consideration both the reuse and safe disposal of waste after treatment, and forming a cycle (from reuse to agriculture to humans) is an ideal solution. The nutrients in excreta can return to the table through agriculture and food production. Hence, with the increasing attention on food security, the investigation of unwanted antibiotics and heavy metals is as important as obtaining the nutrients from human excreta. The control of heavy metal concentrations in human-excreta-related fertilizer is important since heavy metals can accumulate in crops and throughout the food chain, creating a health risk for humans. Heavy metals (such as Cu, Ni, Zn, Cd, Cr, Pb, and Hg) have been detected in solid waste from human excreta (Alvarenga et al., 2015; Remy and Jekel, 2008). Antibiotics are a type of antimicrobial drug used in the treatment and prevention of bacterial infections; they are difficult to degrade, and they can even impart antimicrobial resistance to pathogenic bacteria (Zhu et al., 2017). Hence, the stabilization of heavy metals and elimination of antibiotics are newly-launched hotspots in sanitation research. Nano-filtration, active carbon adsorption, or other advanced treatment processes could be alternative technologies for micro-pollutant removal. Currently, photochemical processes with nanomaterials have been used as new approaches to remove antibiotics and promote antibiotic-resistant microbe removal in wastewater treatment. Materials such as Ag, TiO_2_, and C_3_N_4_ have been studied (Moreira et al., 2016; Nakano et al., 2013; Qu et al., 2013; Thurston et al., 2016), but the practical applications of such technologies for micro-pollutant removal in the sanitation sector are unclear.

## 4 Constraints for global sanitation

Great effort has been made to accelerate the sanitation coverage at different scales to meet the requirements of the MDGs and the upcoming SDG. Nevertheless, gaps in global sanitation still exist in the form of technical, economic, social, cultural, educational, political, and institutional challenges. The previous data show that only 7.9% of the papers on sanitation indexed in the SSCI, indicating a mainstream of technical research and a lack of social study. Nevertheless, it must be mentioned that there has limited publications for SSCI. To realize the targets of SDGs, government support, social acceptability, as well as technological reliability are necessary. From governmental aspects, favorable polices for the sanitation value chain stakeholders (toilet manufacturers, emptying agencies, transportation, treatment system operators, end product sellers as well as users etc.) should be established, such as subsidies, ISO/National standards, regulations, in order to form a healthy market for new sanitation systems, so that everyone could play a specific and positive role in it. Social acceptability is closely linked to private behavior; thus, the developed toilet should adapt to religious belief, ethnic culture, and economic level as well, which would be conducive to governmental motivation. Technological reliability is the driver for sanitation development and practical application. The developed technologies should meet the requirements for safe local discharge, efficient nutrient/energy recovery, limited air emission, non-noise, operability following non-usage/short term/long term shutdown, overload protection, expected lifetime, flexibility in ambient change, etc.

Table 7 lists a brief evaluation of the current techniques described above. The comments for each technology vary according to an overview of the whole “input-operation-output” system. The TRL was also applied using the standards of commercialized applications (such as a centralized treatment plant or decentralized integrated toilet system), where the economic feasibility is distinguished by the development and effective operation of the economic value chain. The relevant policy refers to sustainable market-driven mechanisms to support its development, viability, and up-scaling of the technologies, as well as policies including subsidies, import duties, and taxes from the government. Institutional behavior relies on regular certified laboratory testing to ensure the end-product is safe for use. Social acceptance refers to the acceptance of both the treatment process and end products. Lastly, cultural tradition decides whether the innovative toilet system changes the habits of the users, such as the body-cleaning style, the education/training of individuals, including knowledge dissemination for the stakeholders and users, and the specialized training for workers, administrators, and decision makers. For example, a composting toilet or composting infrastructure incorporating fecal sludge could presently meet the standards for commercial use; the TRL of compost reaches as high as 9. The end products could be used in agriculture. The relevant composting policies and standards have already been established to encourage the creation of organic fertilizer via composting. Therefore, composting meets the criteria for economic feasibility, relevant policy, institutional behavior, and social acceptance. However, the application of a composting toilet would be limited for non-dry toilet users who lack the awareness of its safe management. Additionally, the use of such a toilet requires public education, which would mainly come from the older farming stakeholders. The other 11 techniques have been carefully evaluated through research. These technologies still do not address all the various aspects of sanitation. We found the following challenges and drew a number of conclusions from this work.

**Table 7:** Hotspot-challenge nexus.

Hotspots	TRL level									Economic feasibility	Relevant policy	Institutional behavior	Social acceptance	Cultural tradition	Education/training
	1	2	3	4	5	6	7	8	9						
*Hygienization of excreta*															
Storage									✓	+++	+	−	+	−	−
Alkaline additives				✓						N/A	−	−	−	−	−
Thermal treatment				✓						N/A	−	−	−	−	−

*Resource recovery from excreta*															
MAP/MPP								✓		N/A	−	−	−	−	−
Compost									✓	+++	+	+	+	−	−
Anaerobic digestion									✓	+++	++	++	+	+	+
Ammonia stripping								✓		N/A	−	−	−	−	−
MFC								✓		N/A	−	−	−	−	−
MEC								✓		N/A	−	−	−	−	−

*Micro-pollutants control of excreta*															
Nano filtration				✓						N/A	−	−	−	−	−
Active carbon adsorption				✓						N/A	−	−	−	−	−
Advanced oxidation				✓						N/A	−	−	−	−	−

Notes: 1. TRL Level: TRL (Technology readiness level) are a method of estimating technology maturity of Critical Technology Elements (CTE) of a program during the acquisition process, here was divided into three column, “Research & Development (level 1, 2, 3)”,“Technology Demonstration (level 4, 5, 6, 7)” and “Production & Deployment (level 8, 9)”. TRLs in the European Space Agency: Level 1—Basic principles observed and reported; Level 2—Technology concept and/or application formulated; Level 3—Analytical and experimental critical function and/or characteristic proof-of-concept; Level 4—Component validation in laboratory environment; Level 5—System/subsystem model or prototype demonstration in a laboratory environment; Level 6—System/subsystem model or prototype demonstration in a relevant environment; Level 7—System prototype demonstration in an operational environment; Level 8—Actual system completed and qualified through test and demonstration; Level 9—Actual system proven through successful product launch. 2. Abbreviation: MAP refers to MgNH_4_PO_4_∙6H_2_O; MFC refers to Microbial Fuel Cells; MEC refers to Microbial Electrolysis Cells; MPP refers to MgKPO_4_∙6H_2_O. Marks: “+” refers to the identified technique is satisfied with the corresponding evaluation aspects, the more “+”, the more relevant activities it has. “−” refers to the identified technique is unsatisfied with the corresponding evaluation aspects.

### 4.1 Gaps in technology development and commercialized products

Sanitation issues vary from location to location, season to season, and community to community. People who lack sanitation facilities are often living in the most challenging geographies and climates, so there is no one-fits-all solution that is best for all situations. Different approaches are required for each unique situation (Curry, 2016). Hence, many innovative toilet systems have been developed, especially in regional hotspots. More than one-third of the technologies are at the TRL level 4 (shown in Table 6), which means that most of the new technology is still at the level of component validation in the laboratory. Without three maturing application techniques (a TRL of 9), the rest of the technologies would require further development for practical utilization. Storage techniques for excreta have a positive effect for their hygienization. However, since a large space and long retention time are required for the process, its application is limited in an urban context. The challenges of MAP/MPP precipitation are related to the high recovery efficiency of nutrients and the purification of the products. The purer the products obtained, the more economic value the process will produce. Though MFCs could provide renewable energy during their operation, the high cost of the electrodes might be daunting. Like the MECs, chemical reagents could be replaced by electricity, but the maintenance of the electrodes is crucial. It would be difficult to balance the cost and efficiency at scale. Nano-filtration, active carbon adsorption, and advanced oxidation all require high investment and maintenance costs. New technologies must balance technical effectiveness with economic feasibility.

### 4.2 Gaps in multilateral cooperative solutions: political, institutional, social, cultural, and educational challenges

A survey of the sanitation initiatives in Monkey Bay, a port town on the southern part of Lake Malawi, showed that only 26.6% of the NGO-donated sanitation interventions were in use after two years. This low sustainability was primarily influenced by the lack of consideration of the institutional, technical, educational, operational, social, and cultural factors (Gutierrez, 2007). A study of Timor Tengah Utara (UTT), Nusa Tanagra, Indonesia and the Muong Ang District in northwest Vietnam also showed that the researched districts were areas with high poverty rates, remote households, and a low-density population without access to sewers (Gero et al., 2014; Willetts et al., 2017). Table 6 shows that almost all the mentioned technologies lacked a consideration of social factors. The social, cultural, economic, political, and educational factors have a great effect on the implementation of sanitation solutions (Seetharam, 2015, Uddin et al., 2014, Uddin et al., 2012). Therefore, a functional and coordinated link among these factors can support high-quality sanitation and hygiene services (Rana, 2011).

Government should play a strong leadership role and take responsibility for legislation and the coordination and definition of roles, regulations, and policy. The state should lay a foundation and enforce health standards and regulations. Regulations should create conditions that will be of advantage to innovation both in terms of financing mechanisms and technology support. Products, such as fertilizer, electricity, reclaimed water, biochar, and biodiesel, should be harvested using the most appropriate technology where the operating models, municipal administration, industry, and business model are in demand (Evans et al., 2015). In addition, the institutional support (in the form of re-training, resourcing, and reform) of government and non-government organizations are also of great importance. The relevant cultural and social factors should be taken into account during the planning and execution phases to make sanitation more effective in both urban and rural communities. Therefore, it is important to understand how societies work, including households and communities. Much more consideration should be given to the social, political, and economic institutions that are operating at the local or national level, which include the civil service, schools and colleges, families, and government. It is also necessary to take the various patterns and roles of individuals in societies into consideration and identify who is responsible for the family's health, water supply, and education about defecation habits and environmental hygiene. There is a severe lack of skilled staff that can effectively and efficiently facilitate the sustainable sanitation process, which hampers the continuous follow-up and monitoring of these systems. In addition, an understanding of a community's educational level would help to clarify the issues and solutions for residents and stakeholders to help improve access to adequate sanitation (Gutierrez, 2007). Novel models of institutional, financial, contractual, and legal relationships between communities and agencies should be encouraged (Ademiluyi, 2008). Above all, the improvement of health and sanitation services calls for a good understanding of the actors at various levels, the full involvement and continuous support of the community, and institutional, legal, and contractual linkages among communities, government, and non-government organizations in all stages of the sanitation improvement process (Ekane et al., 2014). To be fully or effective implemented sustainable sanitation, the technologies developed should not only meet the technical standards, but also satisfy the social acceptance, meanwhile, the regulations for the markets and stakeholders behavior, etc. are also important for promoting a sustainable development of sanitation worldwide.

## 5 Conclusions

In this study, bibliometric and word cluster analyses were used to evaluate sanitation research using the Science Citation Index-EXPANDED (SCI-EXPANDED) and the Social Sciences Citation Index (SSCI) from 1992 to 2016. A systematic analysis of global sanitation using the background, current situations, challenges, and perspectives was performed on the results. We demonstrated that researchers are focusing more on sanitation in recent years, which is supported by the increased quantities of publications. Developing countries are facing more serious sanitation problems, but the USA plays a leading role in researching and developing sanitation techniques, ranking first in the quantities of articles and 6 US- innovated toilet systems have been selected from a global pool by BMGF. There are challenges for the adoption of newer technologies in the form of the actual requirements of the people who need them as well as technical development issues. Currently, sanitation encompasses water, solid waste, air pollution, human health, and food security. Hence, a closed-loop analysis of the energy and substance of the internal circulation, transformation, and process control is essential but still lacking in published works. Moreover, although innovative solutions have been developed in terms of the hygienization of human excreta, resource recovery, and removal of micro-pollutants, gaps in the technological development and commercialization of products, as well as issues with integrated solutions that address political, social, institutional, cultural, and educational factors, all still exist. There is no one-size-fits-all approach for achieving the successful implementation of adequate global sanitation. Efforts should be made from view of government support, social acceptability, as well as technological reliability to realize a holistic solution.

## References

[bb0005] Ademiluyi I.A. (2008). Sustainability and impact of community water supply and sanitation programmes in Nigeria: an overview. Afr. J. Agric. Res..

[bb0010] Alvarenga P. (2015). Sewage sludge, compost and other representative organic wastes as agricultural soil amendments: benefits versus limiting factors. Waste Manag..

[bb0015] Andreev N. (2017). Lactic acid fermentation of human urine to improve its fertilizing value and reduce odour emissions. J. Environ. Manag..

[bb0020] Antonini S. (2011). Nitrogen and phosphorus recovery from human urine by struvite precipitation and air stripping in Vietnam. Clean Soil Air Water.

[bb0025] Basakcilardan-Kabakci S. (2007). Recovery of ammonia from human urine by stripping and absorption. Environ. Eng. Sci..

[bb0030] Bell M., Pavitt K. (1993). Technological accumulation and industrial growth: contrasts between developed and developing countries. Ind. Corp. Chang..

[bb0035] Bischel H.N. (2015). Inactivation kinetics and mechanisms of viral and bacterial pathogen surrogates during urine nitrification. Environ. Sci. Water Res. Technol..

[bb0040] Cheng S. (2018). Toilet revolution in China. J. Environ. Manag..

[bb0045] China M.O.F.A. (2015). Report on China's Implementation of the Millennium Development Goals (2000–2015).

[bb0050] Chiu W.T. (2004). Bibliometric analysis of severe acute respiratory syndrome-related research in the beginning stage. Scientometrics.

[bb0055] Chuang K.Y. (2011). High-impact papers presented in the subject category of water resources in the essential science indicators database of the institute for scientific information. Scientometrics.

[bb0060] Curry S. (2016). Five Pressing Global Water & Sanitation Challenges. https://www.cawst.org/blog/bydate/2016/01/five-pressing-global-water-sanitation-challenges/.

[bb0065] Dbira S. (2015). The electrolytic treatment of synthetic urine using DSA electrodes. J. Electroanal. Chem..

[bb0070] Ekane N. (2014). Multi-level sanitation governance: understanding and overcoming challenges in the sanitation sector in sub-Saharan Africa. Waterlines.

[bb0075] Evans B. (2015). VeSV-Value at the End of the Sanitation Value Chain.

[bb0080] Fidjeland J. (2013). The potential for self-sanitisation of faecal sludge by intrinsic ammonia. Water Res..

[bb0085] Fidjeland J. (2015). Modeling the inactivation of ascaris eggs as a function of ammonia concentration and temperature. Water Res..

[bb0090] Finardi U. (2015). Scientific collaboration between BRICS countries. Scientometrics.

[bb0095] Forouzanfar M.H. (2015). Global, regional, and national comparative risk assessment of 79 behavioural, environmental and occupational, and metabolic risks or clusters of risks in 188 countries, 1990–2013: a systematic analysis for the Global Burden of Disease Study 2013. Lancet.

[bb0100] Fu H. (2012). The most frequently cited adsorption research articles in the Science Citation Index (Expanded). J. Colloid Interface Sci..

[bb0105] Fu H. (2013). Mapping of drinking water research: a bibliometric analysis of research output during 1992–2011. Sci. Total Environ..

[bb0115] Fu H. (2014). China's research in chemical engineering journals in Science Citation Index Expanded: a bibliometric analysis. Scientometrics.

[bb0120] Garfield E. (1990). Key-words-plus takes you beyond title words. 2. Expanded journal coverage for current-contents-on-diskette includes social and behavioral-sciences. Curr. Contents.

[bb0125] Gero A. (2014). Relying on markets to address human rights: sanitation supply chain analysis in low-density settings. 37^th^ WEDC International Conference, Hanoi, Vietnam.

[bb0130] Gilbert N. (2009). Environment: the disappearing nutrient. Nat. News.

[bb0135] Gutierrez E. (2007). Delivering pro-poor water and sanitation services: the technical and political challenges in Malawi and Zambia. Geoforum.

[bb0140] Hirsch J.E. (2005). An index to quantify an individual's scientific research output. Proc. Natl. Acad. Sci. U. S. A..

[bb0145] Ho Y. (2010). Japanese lung cancer research trends and performance in Science Citation Index. Intern. Med..

[bb0150] Hoglund C. (1998). Evaluation of faecal contamination and microbial die-off in urine separating sewage systems. Water Sci. Technol..

[bb0155] Hoglund C. (2000). Variation of chemical and microbial parameters in collection and storage tanks for source separated human urine. J. Environ. Sci. Health, Part A: Tox. Hazard. Subst. Environ. Eng..

[bb0160] Hoglund C. (2002). Viral persistence in source-separated human urine. Adv. Environ. Res..

[bb0165] Hoglund C. (2002). Microbial risk assessment of source-separated urine used in agriculture. Waste Manag. Res..

[bb0170] Hou Q. (2015). Mapping the scientific research on life cycle assessment: a bibliometric analysis. Int. J. Life Cycle Assess..

[bb0175] Ieropoulos I.A. (2013). Waste to real energy: the first MFC powered mobile phone. Phys. Chem. Chem. Phys..

[bb0180] Ieropoulos I. (2017). Urine disinfection and in situ pathogen killing using a Microbial Fuel Cell cascade system. PLoS One.

[bb0185] Jadhav U., Hocheng H. (2016). Recovery of phosphorus from source separated urine by *Acidithiobacillus ferrooxidans* culture supernatant. Ecol. Eng..

[bb0190] Kuntke P. (2014). Hydrogen production and ammonium recovery from urine by a Microbial Electrolysis Cell. Int. J. Hydrog. Energy.

[bb0195] Larsen T.A., Gujer W. (1996). Separate management of anthropogenic nutrient solutions (human urine). Water Sci. Technol..

[bb0200] Lazarte M. (2017). Mapping a New Journey for Poo on World Toilet Day. https://www.iso.org/news/ref2243.html.

[bb0205] Ledezma P. (2015). Source-separated urine opens golden opportunities for microbial electrochemical technologies. Trends Biotechnol..

[bb0210] Lewis B. (2017). ISO 30500 to Boost Global Health in Places Without Sewers. https://www.iso.org/news/ref2245.html.

[bb0215] Li W., Zhao Y. (2015). Bibliometric analysis of global environmental assessment research in a 20-year period. Environ. Impact Assess. Rev..

[bb0220] Li J. (2011). Trends in research on global climate change: a Science Citation Index Expanded-based analysis. Glob. Planet. Chang..

[bb0225] Liangyu (2017). Feature: Toilet Revolution to Make India Open Defecation-free. http://news.xinhuanet.com/english/2017-08/14/c_136525422.htm12.21.

[bb0230] Lienert J. (2003). How farmers in Switzerland perceive fertilizers from recycled anthropogenic nutrients (urine). Water Sci. Technol..

[bb0235] Lim S.S. (2012). A comparative risk assessment of burden of disease and injury attributable to 67 risk factors and risk factor clusters in 21 regions, 1990–2010: a systematic analysis for the Global Burden of Disease Study 2010. Lancet.

[bb0245] Liu B. (2015). Air stripping process for ammonia recovery from source-separated urine: modeling and optimization. J. Chem. Technol. Biotechnol..

[bb0250] Liu H. (2017). Simultaneous pollutant removal and electricity generation in a combined ABR-MFC-MEC system treating fecal wastewater. Water Air Soil Pollut..

[bb0255] Magri M.E. (2013). Inactivation of pathogens in feces by desiccation and urea treatment for application in Urine-Diverting Dry Toilets. Appl. Environ. Microbiol..

[bb0260] Magri M.E. (2015). Inactivation of adenovirus, reovirus and bacteriophages in fecal sludge by pH and ammonia. Sci. Total Environ..

[bb0265] Mahmood I.B. (2015). Co-composting of fecal matter in Mongolia using two different technologies. J. Water Sanitation Hyg. Dev..

[bb0270] Mao N. (2010). A bibliometric study of the trend in articles related to risk assessment published in Science Citation Index. Hum. Ecol. Risk. Assess..

[bb0275] Maurer M. (2006). Treatment processes for source-separated urine. Water Res..

[bb0280] Mehta N. (2014). Combating the ‘Toilet Challenge’ With Science. http://www.livemint.com/Politics/9VKLhtq2Eti8yU20HPoQeN/Combating-the-toilet-challenge-with-science.html.

[bb0285] Merino-Jimenez I. (2017). Enhanced MFC power production and struvite recovery by the addition of sea salts to urine. Water Res..

[bb0290] Mihelcic J.R. (2011). Global potential of phosphorus recovery from human urine and feces. Chemosphere.

[bb0295] Moreira N.F.F. (2016). Photocatalytic ozonation of urban wastewater and surface water using immobilized TiO_2_ with LEDs: micropollutants, antibiotic resistance genes and estrogenic activity. Water Res..

[bb0300] Mulec A.O. (2016). Composting of the solid fraction of blackwater from a separation system with vacuum toilets - effects on the process and quality. J. Clean. Prod..

[bb0305] Murray C.J., Lopez A.D. (1997). Global mortality, disability, and the contribution of risk factors: Global Burden of Disease Study. Lancet.

[bb0310] Nakano R. (2013). Broad spectrum microbicidal activity of photocatalysis by TiO_2_. Catalysts.

[bb0315] Nancharaiah Y.V. (2016). Recent advances in nutrient removal and recovery in biological and bioelectrochemical systems. Bioresour. Technol..

[bb0320] Niwagaba C. (2009). Comparing microbial die-off in separately collected faeces with ash and sawdust additives. Waste Manag..

[bb0325] Niwagaba C. (2009). Bench-scale composting of source-separated human faeces for sanitation. Waste Manag..

[bb0330] Nooy W.D. (2011). Exploratory Social Network Analysis With Pajek.

[bb0335] Nordin A. (2013). Pathogen and indicator inactivation in source-separated human urine heated by the sun. J. Water Sanitation Hyg. Dev..

[bb0340] World Health Organization (WHO) (2006). Guidelines for the safe use of wastewater, excreta and greywater: wastewater and excreta use in aquaculture v. 3. Water Sanitation Health.

[bb0345] Osseiran N. (2017). 2.1 Billion People Lack Safe Drinking Water at Home, More Than Twice As Many Lack Safe Sanitation. http://www.who.int/mediacentre/news/releases/2017/water-sanitation-hygiene/en/.

[bb9000] Persson O. (1994). The intellectual base and research fronts of JASIS 1986–1990. J. Am. Soc. Inf. Sci..

[bb0350] Persson O. (2009). How to use Bibexcel for various types of bibliometric analysis. Celebrating Scholarly Communication Studies A Festschrift for Olle Persson at His Birthday.

[bb0355] Pradhan S.K. (2017). Nitrogen and phosphorus harvesting from human urine using a stripping, absorption, and precipitation process. Environ. Sci. Technol..

[bb0360] PRC, N.H.A.F (2016). China Health Statistical Yearbook, 1950–2015.

[bb0365] Qian F. (2015). A bibliometric analysis of global research progress on pharmaceutical wastewater treatment during 1994–2013. Environ. Earth Sci..

[bb0370] Qu X. (2013). Applications of nanotechnology in water and wastewater treatment. Water Res..

[bb0375] Rana M.M.P. (2011). Urbanization and sustainability: challenges and strategies for sustainable urban development in Bangladesh. Environ. Dev. Sustain..

[bb0380] Remy C., Jekel M. (2008). Sustainable wastewater management: life cycle assessment of conventional and source-separating urban sanitation systems. Water Sci. Technol..

[bb0385] Rodriguez Arredondo M. (2017). Load ratio determines the ammonia recovery and energy input of an electrochemical system. Water Res..

[bb0390] Ronteltap M. (2007). Struvite precipitation thermodynamics in source-separated urine. Water Res..

[bb0395] Salar-Garcia M.J. (2017). Electricity production from human urine in ceramic microbial fuel cells with alternative non-fluorinated polymer binders for cathode construction. Sep. Purif. Technol..

[bb0400] Seetharam K. (2015). Challenges and opportunities for sanitation in developing countries. J. Sci. Pol. Gov..

[bb0405] Sossou S.K. (2014). Inactivation mechanisms of pathogenic bacteria in several matrixes during the composting process in a composting toilet. Environ. Technol..

[bb0410] Tan J. (2014). A bibliometric analysis of research on proteomics in Science Citation Index Expanded. Scientometrics.

[bb0415] Thurston J.H. (2016). Preparation and characterization of photoactive antimicrobial graphitic carbon nitride (g-C_3_N_4_) films. RSC Adv..

[bb0420] Tilley E. (2008). Effects of storage on phosphorus recovery from urine. Environ. Technol..

[bb0425] Uddin S.M.N. (2012). Assessment of social acceptance and scope of scaling up urine diversion dehydration toilets in Kenya. J. Water Sanitation Hyg. Dev..

[bb0430] Uddin S.M.N. (2014). Socio-cultural acceptance of appropriate technology: identifying and prioritizing barriers for widespread use of the urine diversion toilets in rural Muslim communities of Bangladesh. Technol. Soc..

[bb0435] UNICEF (2015). Progress on Sanitation and Drinking Water: 2015 Update and MDG Assessment.

[bb0440] UNICEF (2016). The State of the World's Children.

[bb0445] Vinneras B. (2007). Comparison of composting, storage and urea treatment for sanitising of faecal matter and manure. Bioresour. Technol..

[bb0450] Vinneras B. (2008). Inactivation of bacteria and viruses in human urine depending on temperature and dilution rate. Water Res..

[bb0455] Wang M. (2015). Global trends in soil monitoring research from 1999 to 2013: a bibliometric analysis. Acta Agric. Scand..

[bb0460] Wang W. (2017). Catalytic liquefaction of municipal sewage sludge over transition metal catalysts in ethanol-water co-solvent. Bioresour. Technol..

[bb0465] Watch W. (2016). Brazil. https://www.washwatch.org/en/countries/brazil/summary/statistics/.

[bb0470] WaterAid (2016). Overflowing Cities: The State of the Word's Toilets.

[bb0475] WHO (2015). Progress on Sanitation and Drinking Water: 2015 Update and MDG Assessment.

[bb0480] Willetts J. (2017). Sanitation value chains in low density settings in Indonesia and Vietnam: impetus for a rethink to achieve pro-poor outcomes. J. Water Sanitation Hyg. Dev..

[bb0485] Wilsenach J.A. (2007). Phosphate and potassium recovery from source separated urine through struvite precipitation. Water Res..

[bb0490] Wong M.H. (2017). China to Get Extreme Toilet Makeover to Boost Tourism. http://www.cnn.com/travel/article/china-toilet-revolution/index.html.

[bb0495] Worley H. (2014). Water, Sanitation, Hygiene, and Malnutrition in India. http://www.prb.org/Publications/Articles/2014/india-sanitationmalnutrition.aspx.

[bb0500] Xu K. (2012). Laboratory experiments on simultaneous removal of K and P from synthetic and real urine for nutrient recycle by crystallization of magnesium-potassium-phosphate-hexahydrate in a draft tube and baffle reactor. Chemosphere.

[bb0505] Xu K. (2015). The precipitation of magnesium potassium phosphate hexahydrate for P and K recovery from synthetic urine. Water Res..

[bb0510] Yin Z. (2018). Sludge disinfection using electrical thermal treatment: the role of ohmic heating. Sci. Total Environ..

[bb0515] Yuan P., Kim Y. (2017). Increasing phosphorus recovery from dewatering centrate in microbial electrolysis cells. Biotechnol. Biofuels.

[bb0520] Zang G. (2012). Nutrient removal and energy production in a urine treatment process using magnesium ammonium phosphate precipitation and a microbial fuel cell technique. Phys. Chem. Chem. Phys..

[bb0525] Zhang G. (2010). A bibliometric analysis of world volatile organic compounds research trends. Scientometrics.

[bb0530] Zhang B. (2014). A bibliometric analysis of research on upflow anaerobic sludge blanket (UASB) from 1983 to 2012. Scientometrics.

[bb0535] Zhang C. (2017). Recovery of phosphorus and potassium from source-separated urine using a fluidized bed reactor: optimization operation and mechanism modeling. Ind. Eng. Chem. Res..

[bb0540] Zheng T. (2017). Benchmarking the scientific research on wastewater-energy nexus by using bibliometric analysis. Environ. Sci. Pollut. Res..

[bb0545] Zheng T. (2018). State of the art on granular sludge by using bibliometric analysis. Appl. Microbiol. Biotechnol..

[bb0550] Zhou X. (2017). Investigation on microbial inactivation and urea decomposition in human urine during thermal storage. J. Water Sanitation Hyg. Dev..

[bb0555] Zhu Y. (2017). Microbial mass movements. Science.

[bb0560] Zollig H. (2017). Removal rates and energy demand of the electrochemical oxidation of ammonia and organic substances in real stored urine. Environ. Sci. Water Res. Technol..

